# Integration of Transcriptome Profiling and Single‐Cell Sequencing Analysis to Establish a CD8+ T Cell–Related Prognostic Model for Patients With NSCLC: From Assessment to Therapy

**DOI:** 10.1002/cam4.71337

**Published:** 2025-11-12

**Authors:** Yi‐yang Jiang, Min‐min Yu, Xia Cui, Xue Li, Bin‐bin Li, Jing‐tao Zhang, Fei Xu

**Affiliations:** ^1^ The First School of Clinical Medicine Shandong University of Traditional Chinese Medicine Jinan China; ^2^ Department of Pathology Affiliated Hospital of Shandong University of Traditional Chinese Medicine Jinan China; ^3^ Department of Neurology Affiliated Hospital of Shandong University of Traditional Chinese Medicine Jinan China; ^4^ School of Acupuncture and Tuina Shandong University of Traditional Chinese Medicine Jinan China; ^5^ The Second School of Clinical Medicine Guangzhou University of Traditional Chinese Medicine Guangzhou China; ^6^ Department of Respiratory and Critical Care Medicine Affiliated Hospital of Shandong University of Traditional Chinese Medicine Jinan China

**Keywords:** biomarker, CD8^+^ T cell, drug sensitivity, nomogram, non‐small‐cell lung cancer

## Abstract

**Background:**

Non‐small cell lung cancer (NSCLC) is the leading cause of cancer‐related mortality, characterized by a poor prognosis. The advent of immunotherapy has significantly altered the treatment landscape for NSCLC. CD8^+^ T cells, key mediators of immune responses, play a pivotal role in the prognosis and progression of the disease. This study aims to develop a CD8^+^ T‐cell‐related prognostic model to enable more precise prognostic evaluations and enhance clinical decision‐making in immunotherapy for patients with NSCLC.

**Methods:**

Three datasets (TCGA‐NSCLC, GSE183219, and GSE42127) were analyzed using Weighted Gene Co‐expression Network Analysis and single‐cell analysis. A risk model was constructed through least absolute shrinkage and selection operator, univariate Cox regression, and multivariate Cox regression analyses. A prognostic nomogram was subsequently developed, integrating the risk model and clinical characteristics of patients with NSCLC, and validated using multiple methods. Additionally, Gene Set Enrichment Analysis, immune‐related analyses, and drug susceptibility assays were performed to assess responses to immunotherapy and chemotherapy.

**Results:**

*CD52*, *CD69*, and *PLIN2* were identified as biomarkers and used to construct a risk model with high accuracy. Based on the risk model, patients were classified into high‐ and low‐risk subgroups. The model demonstrated strong predictive performance in both the training and validation cohorts. When combined with pathologic N and T stages, a clinical prognostic nomogram was developed, outperforming individual indicators in prognostic prediction. Immune landscape analyses revealed a robust immune system in the low‐risk group, whereas immune dysfunction was observed in the high‐risk group, suggesting differential immunotherapy efficacy between the cohorts. Additionally, paclitaxel showed significantly greater effectiveness in the high‐risk group.

**Conclusion:**

This study constructed an innovative CD8^+^ T cell‐related risk model, advancing clinical diagnosis and offering valuable therapeutic strategies for patients with NSCLC.

## Graphical abstract

CD52, CD69, and PLIN2 were identified as potential biomarkers in NSCLC. Immune‐related pathways were enriched and immune system dysfunction occurred. Paclitaxel was significantly more effective in the high‐risk group.

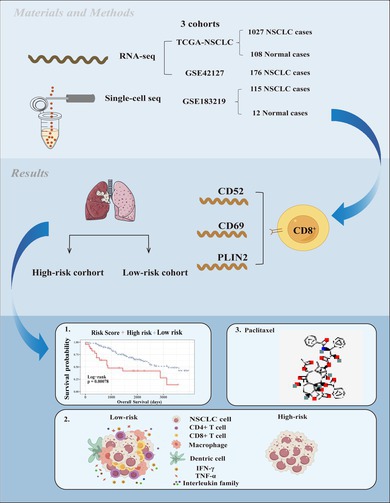

**Abbreviations**
APC: antigen‐presenting cell
AUC: area under the curve
CTLA‐4: cytotoxic T‐lymphocyte‐associated protein 4
CYT: cytolytic activity score
DCA: decision curve analysis
DCs: dendritic cells
DEG: differentially expressed gene
EGFR‐TKI: epidermal growth factor receptor‐tyrosine kinase inhibitor
EMT: epithelial‐mesenchymal transition
GEO: gene expression omnibus
GO: gene ontology
GSEA: gene set enrichment analysis
IC50: Half maximal inhibitory concentration
ICIs: immune checkpoint inhibitors
JAK1: Janus kinase 1
KEGG: Kyoto Encyclopedia of Genes and Genomes
KM: Kaplan–Meier
LASSO: least absolute shrinkage and selection operator
LUAD: lung adenocarcinoma
LUSD: lung squamous cell carcinoma
MHC: major histocompatibility complex
NK: natural killer
NSCLC: non‐small cell lung cancer
OS: overall survival
PCA: principal component analysis
PD‐1: programmed cell death protein 1
PD‐L1: programmed death‐ligand 1
ROC: receiver operating characteristic
ssGSEA: single‐sample gene set enrichment analysis
STAT1: signal transducer and activator of transcription 1
TCGA: The Cancer Genome Atlas
TCR: T‐cell receptor
TIME: tumor immune microenvironment
TME: tumor microenvironment
VEGF: vascular endothelial growth factor
WGCNA: weighted gene co‐expression network analysis

## 1 Introduction

Non‐small‐cell lung cancer (NSCLC) accounts for 85% of all cases of lung cancer which is the primary cause of global cancer‐related death associated with high rates of illness and death [1]. The limited advancements in clinical cure rates, coupled with a 5‐year survival rate of only 26.4% [2], drive efforts toward the development of new treatment strategies for NSCLC. Recent findings have revealed the complex dynamics within the tumor immune microenvironment (TIME). Immunotherapy has emerged as a promising therapeutic approach and is increasingly utilized as a first‐line treatment for select individuals with metastatic NSCLC. However, while many patients with NSCLC exhibit favorable clinical responses to anti‐tumor immunotherapy, a significant proportion fail to benefit. Identifying populations most likely to benefit from immunotherapy is critical for optimizing clinical decision‐making.

CD8^+^ T cell is the predominant subtype of T cells participating in the antitumor response within the TME and drives the immunotherapy response [3]. Thus, its abundance, functional status, as well as spatial distribution hold promise as dependable prognostic indicators for patients with NSCLC. A retrospective study on the immune status of the local tumor microenvironment in NSCLC tissues discovered that patients with higher densities of CD8^+^ T cells and DC cells in their resection tissues exhibited better prognoses [4]. The increased tumor‐infiltrating lymphocytes, especially CD3^+^CD8^+^ T cells, were proved associated with improved prognosis of NSCLC [5]. These indicate that there is a direct connection between CD8^+^ T cells and NSCLC prognosis. Furthermore, the expression of CD8^+^ T cells changes significantly before and after NSCLC treatment, which also indicates that the function of CD8^+^ T cells is significantly associated with disease progression [6]. Not only that, an analysis conducted on the primary tumors and lymph nodes of 271 NSCLC patients found that low proximity of PD‐1^+^TIM‐3^−^CD8^+^ tissue‐resident memory T (T_RM2_) cells to tumor cells was associated with the recurrence of early lung squamous cell carcinoma (LUSD), as well as the high proximity of PD‐1^+^TIM‐3^+^CD8^+^ T_RM4_ cells to tumor tissues implicated the increased recurrence of early lung adenocarcinoma (LUAD) [6]. Concurrently, the small percentage of normal CD8^+^ T cells in the tumor center and the substantial population of dysfunctional CD8^+^ T cells on the invasive margin exhibit inverse correlation with prognosis [7]. These studies emphasized the importance of spatial distribution and functional states of CD8^+^ T cells in NSCLC prognosis. Thus, it follows that CD8^+^ T cells occupy a powerful position in predicting NSCLC outcomes.

CD8^+^ T cells are widely distributed within NSCLC, where they are typically activated by antigen‐presenting cells (APCs), release inflammatory mediators, and specifically target and eliminate cancer cells. These cells also respond favorably to immunotherapies such as PD‐1 blockade therapy [8]. The antitumor function of CD8^+^ T cells is not an isolated process but depends on synergistic interactions with other immune cells in the tumor microenvironment (TME). For instance, their activation relies on antigen presentation by dendritic cells (DCs); dysfunctional DCs in NSCLC lead to impairments in CD8^+^ T cells, thereby weakening their antitumor activity [9]. Additionally, natural killer (NK) cells can enhance the specific responses of CD8^+^ T cells through cross‐presentation of tumor antigens, and the co‐infiltration of NK cells and CD8^+^ T cells in NSCLC is an independent predictor of favorable prognosis [10]. These interactions collectively form the immunological network basis for CD8^+^ T cells to exert antitumor functions, and also render the prognostic value of their biomarkers more systematic [11].

The anti‐tumor activities of CD8^+^ T cells are affected significantly by their surface biomarkers. Long‐term stimulation by a chronic inflammatory environment leads to CD8^+^ T cell exhaustion and dysfunction [12], manifested by high expression of PD‐1 and the transcription factor TOX. This depletion programme decreased tumor sensitivity to immunotherapy and PD‐1 blockade only partially reverses this state [13, 14]. PD‐1 blockade only partially reverses this state. TIM‐3 is a marker of terminally depleted T cells, co‐expressed with PD‐1 in TME, and its upregulation is associated with immunotherapy resistance [15]. Through the TOX‐dependent LAG‐3/TOX/NKG2A axis, LAG‐3 diminishes CD8^+^ T cell function and cytokine secretion [16]. CD39^+^CD8^+^ T cells suppress the immune response by hydrolysing ATP to generate adenosine in tumors [17]. Inhibition of CD39/CD73 reverses immunosuppression and enhances the PD‐1 therapeutic response [18]. This prognostic‐therapeutic response correlation allows CD8^+^ T‐cell‐associated biomarkers to not only predict prognosis but also to explain the immunological mechanisms of prognostic differences. Nevertheless, the regulatory genes in CD8^+^ T cells associated with cancer development remain under explored.

Previous studies have demonstrated that high PD‐1 antigen expression on exhausted CD8^+^ T cells is associated with improved survival in patients treated with PD‐1 blockade in NSCLC [19]. However, a comprehensive and reliable model to predict which populations are more likely to respond to immunotherapy remains lacking. Although traditional TNM staging can reflect tumor burden, it struggles to capture the heterogeneity of the immune microenvironment, whereas CD8^+^ T cell‐related biomarkers can effectively identify such high‐risk populations [20]. Additionally, dynamic changes in CD8^+^ T cell subsets provide a more precise basis for prognostic stratification [6], which further highlights the unique value of CD8^+^ T cell biomarkers in prognostic assessment. Therefore, further investigation of CD8^+^ T cell‐related biomarkers and the development of a robust risk model are essential to enhance diagnosis, treatment, and prognostic prediction in NSCLC.

In this study, data from three NSCLC‐related datasets (TCGA‐NSCLC, GSE183219, and GSE42127) were utilized. The TCGA‐NSCLC dataset was employed for primary analysis, GSE183219 was used for single‐cell sequencing analysis, and GSE42127 served as the validation dataset. Through comprehensive bioinformatics analyses, CD8^+^ T cell‐related biomarkers were identified to construct a risk model. This model stratified patients into two subgroups based on risk scores, which were significantly associated with differences in overall survival (OS). A clinical prognostic model, incorporating clinical features of NSCLC, was developed to assess patient outcomes. Additionally, the predictive value of the risk model was explored through immune landscape and drug sensitivity analyses. This study provides valuable insights to support clinical decision‐making in NSCLC.

## 2 Materials and Methods

### 2.1 Acquiring and Processing of Data

Data on LUSD and LUAD were extracted from the Cancer Genome Atlas (TCGA)‐NSCLC dataset using UCSC Xena (https://xenabrowser.net/datapages/). To construct and evaluate the risk model, a training cohort consisting of 1027 NSCLC samples and 108 control samples from the TCGA‐NSCLC dataset was analyzed. Additionally, the GSE42127 and GSE183219 datasets were retrieved from the Gene Expression Omnibus (GEO) database (https://www.ncbi.nlm.nih.gov/geo/) using the R package “GEOquery” (version 2.62.2) [21]. The GSE42127 dataset, comprising 176 patients with NSCLC, was used as the validation cohort to assess the accuracy of the risk model. Microarray sequencing data were obtained using the GPL6884 Illumina HumanWG‐6 v3.0 expression beadchip platform [22]. The GSE183219 dataset contains single‐cell high‐throughput sequencing data from 15 NSCLC and 12 normal samples, sequenced using the GPL18573 Illumina NextSeq 500 platform [23]. By excluding samples with missing clinical information from the datasets, only those with complete survival information and clinical data were retained. The clinical information specifically included age, gender, Stage, M_stage, N_stage, and T_stage. Detailed information on samples in the TCGA‐NSCLC and GSE42127 datasets was provided in Tables 1 and 2.

**TABLE 1:** Descriptive statistics of basic information in TCGA‐NSCLC dataset.

Characters	Counts
Age, years (mean ± SD)	66.06 ± 9.42
Gender, *n* (%)	
Female	380 (40%)
Male	570 (60%)
TNM stage, *n* (%)	
I	489 (51.5%)
II	265 (27.9%)
III	155 (16.3%)
IV	30 (3.2%)
Unknown	11 (1.2%)
T stage	
T1	269 (28.3%)
T2	531 (55.9%)
T3	110 (11.6%)
T4	37 (3.9%)
Tx	3 (0.3%)
N stage	
N0	611 (64.3%)
N1	212 (22.3%)
N2	103 (10.8%)
N3	7 (0.7%)
Nx	17 (1.8%)
M stage	
M0	707 (74.4%)
M1	29 (3.1%)
Mx	214 (22.5%)
Overall survival, days Median (95% CI)	701 (658,759)

**TABLE 2:** Descriptive statistics of basic information in GSE42127 dataset.

Characteristic	Counts
Type, *n* (%)	
LUAD	133 (75.6%)
LUSD	43 (24.4%)
Age, years (mean ± SD)	65.96 ± 10.92
Gender, *n* (%)	
Female	83 (47.2%)
Male	93 (52.8%)
TNM stage, *n* (%)	
I	112 (63.6%)
II	32 (18.2%)
III	30 (17.0%)
IV	1 (0.6%)
Unknown	1 (0.6%)
Overall survival, days Median (95% CI)	1422 (1188, 1548)

### 2.2 Identification of Genes Differentially Expressed in NSCLC

Differentially expressed genes (DEGs) were identified between NSCLC and healthy tissue samples in the TCGA‐NSCLC dataset using the R package “DESeq2” (version 1.34.0) [24]. DEGs were selected based on the criteria of a *p*.adjust value < 0.05 and |log_2_FC| > 1. A volcano plot was then generated using the “ggplot2” package (version 3.3.5) [25]. Additionally, a heatmap displaying the top 10 dysregulated genes was created using the “ComplexHeatmap” package (version 2.10.0) [26].

### 2.3 Identifying and Analyzing CD8 ^+^ T Cell‐Associated Genes

Immune cell scores, representing cell abundance in the TCGA‐NSCLC dataset, were computed using the R package “xCell” [27]. To investigate the influence of CD8^+^ T cell abundance on OS in patients with NSCLC, Kaplan–Meier (KM) survival analysis was performed using the “survival” R package (version 3.3‐1) [28] and the “survminer” R package (version 0.4.9) [29]. Two categories were defined based on an optimal CD8^+^ T cell score threshold, followed by KM analysis. Furthermore, module genes strongly associated with CD8^+^ T cells were selected using Weighted Gene Co‐expression Network Analysis (WGCNA) with the R package “WGCNA” (version 1.70‐3) [30].

### 2.4 Single‐Cell Sequencing Data Analysis and Acquisition of DEGs in CD8 ^+^ T Cells

The single‐cell GSE183219 dataset retrieved from the GEO database was preprocessed and normalized using the “Seurat” R package (version 4.1.0) [31]. The following cut‐off criteria were applied: gene counts within each cell ranged from 100 to 3500, the total number of molecules was < 10,000, and the proportion of mitochondrial genes was < 5%. Next, the FindVariableFeatures function was used to screen for significantly differentially expressed genes across cells. Subsequently, the JackStraw method, principal component analysis (PCA), and UMAP algorithm were applied for dimensionality reduction and clustering analysis to obtain distinct cell clusters. Differential analysis for each cell cluster was then performed using the FindAllMarkers function (with parameters: min.pct ≥ 0.25, logfc.threshold = 0.5, test.use = “wilcox”). Marker genes were ranked by statistical significance and effect size for each cluster, and the top 1 gene was selected as the representative marker of the cluster. Cell type mapping was then conducted using the “SingleR” R package (version 1.831) [32], and annotation results were visualized in UMAP space using the DimPlot function. The labels from SingleR were cross‐validated with manually curated markers (top markers from DotPlot): if the two were consistent, the annotation was adopted directly; if there was ambiguity, the annotation was re‐evaluated by integrating classical markers and literature [33, 34]. In addition, the abundance distribution of various cell types in the NSCLC group and control group was analyzed. DEGs in CD8^+^ T cells between NSCLC samples and control samples were screened using the FindMarkers function of the “Seurat” R package, which were used for the selection of prognostic genes.

### 2.5 Establishment and Validation of the Risk Model

Candidate genes were obtained by taking the intersection of three gene sets: CD8^+^ T cell‐related DEGs from the single‐cell RNA sequencing dataset GSE183219, CD8^+^ T cell module genes identified by WGCNA analysis, and DEGs from the bulk RNA‐seq dataset TCGA‐NSCLC. Univariate and multivariate Cox regression analyses, as well as Least Absolute Shrinkage and Selection Operator (LASSO) regression analysis, were performed using the “glmnet” (version 4.1‐2) [35] and “survival” R packages to filter the marker genes. The resulting risk model was constructed as follows: risk score = $\sum_{1}^{n}\left(\text{coefficient}\left(\text{genes}\right)\times \text{expression}\left(\text{genes}\right)\right)$.

Validation was performed using NSCLC sample data from the TCGA‐NSCLC and GSE42127 datasets. First, the risk scores of NSCLC samples were calculated by integrating candidate genes and their coefficients. Samples with survival information in the TCGA‐NSCLC and GSE42127 datasets were divided into high‐risk and low‐risk groups based on the optimal risk score cutoff. Subsequently, KM survival analysis was used to characterize the survival status of high‐risk versus low‐risk patients. The “survivalROC” package (version 1.0.3) was applied to assess the accuracy of survival prediction via receiver operating characteristic (ROC) analysis, with the area under the curve (AUC) > 0.6 indicating favorable predictive performance [36]. Finally, heatmaps were generated to visualize the expression patterns of prognostic genes in high‐risk and low‐risk groups across both datasets. Survival analysis, ROC analysis, and expression analysis of prognostic genes in high/low‐risk groups were performed for both the TCGA‐NSCLC and GSE42127 datasets to validate the predictive performance of the model.

### 2.6 Establishing and Estimating a Clinical Prognostic Model

Clinical data, including pathological M, N, and T stages, age, sex, and tumor stage, were extracted from the TCGA‐NSCLC dataset and assigned respective risk scores. Univariate and multivariate Cox regression analyses were performed using the “survival” R package to identify independent prognostic factors. Factors with *p‐*values < 0.05 in both models were considered significant. A nomogram was constructed to predict the risk of mortality in patients with NSCLC based on these independent prognostic factors, and its accuracy was validated through calibration curves, ROC analysis, and Decision Curve Analysis (DCA) using the “rms” R package (version 6.2‐0) [37].

### 2.7 Functional Enrichment Analyses

The functional roles of DEGs, module genes, CD8^+^ T cell‐related genes, and candidate genes were explored using the “clusterProfiler” package. Gene Ontology (GO) and Kyoto Encyclopedia of Genes and Genomes (KEGG) analyses were conducted to investigate the enriched biological processes associated with module genes [38].

To assess the enriched pathways related to the risk model, Gene Set Enrichment Analysis (GSEA) was performed on genes from both groups, using the HALLMARK gene set for functional enrichment analysis. Additionally, the KEGG gene set was employed for single‐sample GSEA (ssGSEA) to identify pathways associated with the risk score. The “GSVA” R package (version 1.42.0) [39] was used for this analysis.

### 2.8 Analyzing the Immune Landscape and Drug Sensitivity

Analysis of the TCGA‐NSCLC data using ssGSEA identified 28 cell types and 17 relevant pathways. The MCP‐counter technique was employed to estimate the proportions of 10 immune cell types in each group, enabling a reliable comparison. Cytolytic activity (CYT), along with the expression of PRF1 and GZMA, was used to assess CD8^+^ T cell activity. Additionally, expression analyses of co‐stimulatory molecules, major histocompatibility complexes (MHCs), chemokines and receptors, and 48 immune checkpoints were conducted across the two risk categories to evaluate the immune response in NSCLC.

The effectiveness of five NSCLC chemotherapeutics—erlotinib, paclitaxel, gemcitabine, imatinib, and cisplatin—was assessed in the established risk categories from the TCGA‐NSCLC dataset. The “oncoPredict” program (version 0.1) was used to determine the half maximal inhibitory concentration (IC50) values for each NSCLC specimen [40]. Drug response was reflected in the IC50 values, with lower IC50 values indicating stronger drug efficacy.

### 2.9 Statistical Analysis

All statistical analyses were performed using the R software package. A *p*‐value of < 0.05 was considered statistically significant. The Wilcoxon test was used for comparisons between two groups, and the Kruskal‐Wallis test was applied for comparisons involving more than two groups.

## 3 Results

### 3.1 Identification and Analysis of NSCLC‐Associated DEGs

**FIGURE 1:**
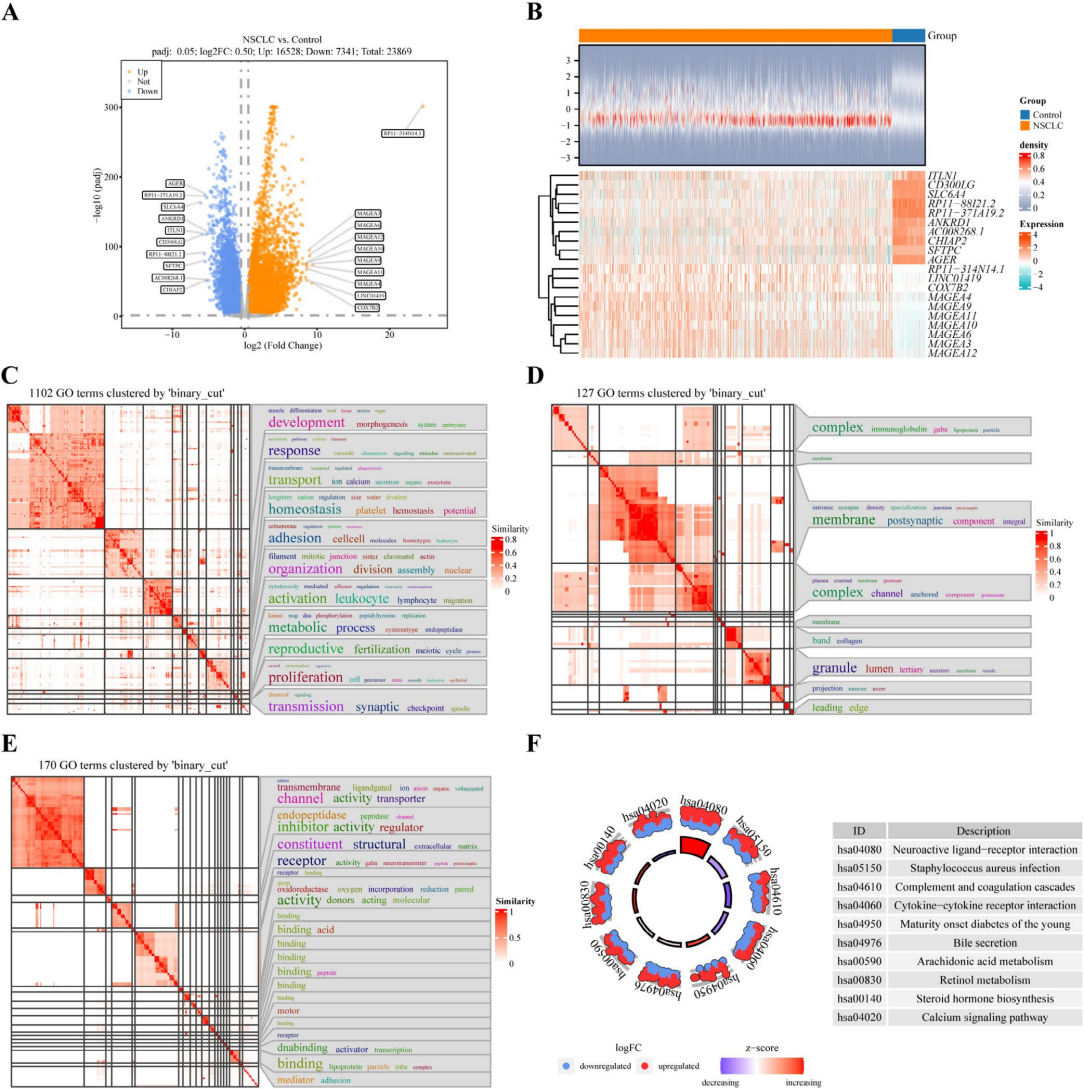
Identification of DEGs and functional enrichment analysis. (A) Volcano plot of DEGs comparing NSCLC and normal tissues. Upregulated genes are represented by red dots, and downregulated genes by blue spots. (B) Heatmap displaying the distribution of the top 10 upregulated and downregulated genes between the two groups. (C–F) Functional enrichment analysis for DEGs. BP (C), CC (D), and MF (E) are presented as system cluster diagrams. The top 10 KEGG pathways are shown in a Bagua map (F).

A total of 23,869 DEGs were identified from the extracted samples. Among these, 16,528 genes were upregulated, and 7341 were downregulated in NSCLC samples (Table S1, Figure 1A). The top 10 most significant DEGs, including upregulated MAGE family genes, which are highly specific to lung cancer, and downregulated *SFTPC*, crucial for maintaining normal lung function, were visualized as a heatmap (Figure 1B). GO analysis of the DEGs revealed enrichment in processes related to cell proliferation, membrane components, and other cellular constituents (Figure 1C–E). KEGG analysis highlighted pathways associated with metabolism, including bile secretion, arachidonic acid metabolism, and retinol metabolism (Figure 1F).

### 3.2 Module Genes Were Identified and Analyzed Through WGCNA

**FIGURE 2:**
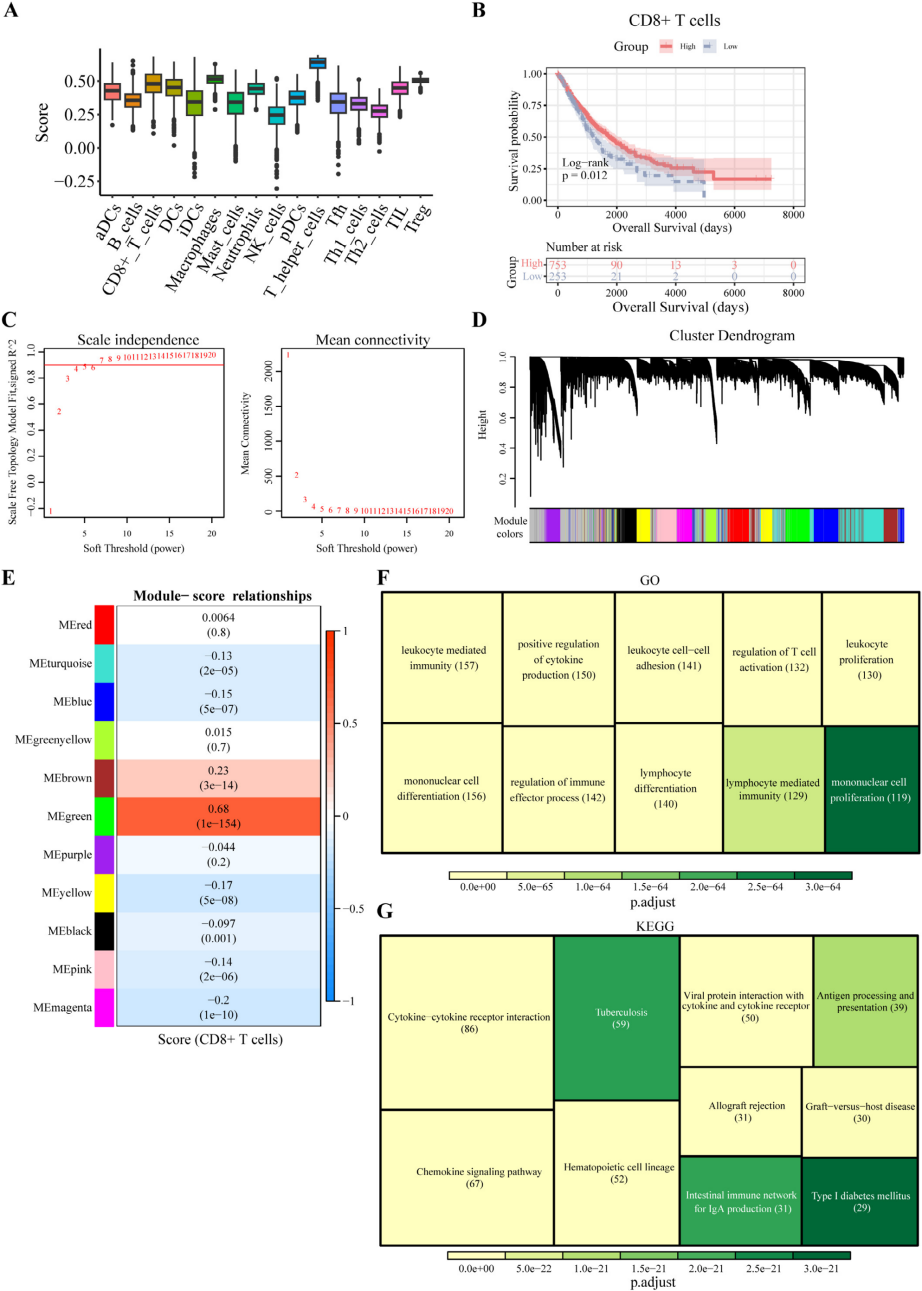
WGCNA and biological function analysis of module genes. (A) Box plot depicting immune cell abundance. The *x‐*axis represents immune cell types, and the *y*‐axis shows the cell abundance values. (B) Kaplan–Meier curves demonstrating that overall survival (OS) was significantly shorter in patients with low CD8^+^ T cell infiltration compared to those with high CD8^+^ T cell infiltration (*p* = 0.012). (C) Evaluation of mean connectivity (right) and scale‐free fit index (left) for distinct soft threshold powers. (D) Cluster dendrogram of DEGs resulting in 11 non‐gray modules. (E) Heatmap of the correlation between modules and CD8^+^ T cell score. (F, G) GO and KEGG functional enrichment analyses of module genes, visualized as rectangular dendrograms, showing the top 10 pathways.

To identify genes strongly correlated with CD8^+^ T cells in TCGA‐NSCLC, the abundance scores of 16 immune cell types were calculated using the xCell method. T helper cells exhibited the highest abundance score, while natural killer (NK) cells showed the lowest score (Figure 2A). CD8^+^ T cells displayed significant infiltration. Using an optimal threshold score for CD8^+^ T cells (0.4125346), two subgroups were established, representing patients with NSCLC exhibiting high and low CD8^+^ T cell abundance. KM survival analysis indicated that patients with higher CD8^+^ T cell abundance had significantly better OS (Figure 2B). This also suggested a lower incidence of immune evasion in these patients, allowing CD8^+^ T cells to effectively exert their anti‐cancer effects.

WGCNA identified 1149 module genes strongly associated with CD8^+^ T cells. A soft threshold power of seven was used to construct a scale‐free network, achieving an *R* ^2^ value of 0.9 (Figure 2C). Eleven non‐gray modules were identified and visualized in a gene dendrogram (Figure 2D). Correlation analysis of CD8^+^ T cells with module scores revealed that the green module was positively correlated with CD8^+^ T cells (cor = 0.68) (Figure 2E). GO and KEGG analyses of the module genes indicated significant associations with immune responses and regulation of immune cells (Figure 2F,G).

### 3.3 CD8 ^+^ T Cells in the Single‐Cell GSE183219 Dataset

**FIGURE 3:**
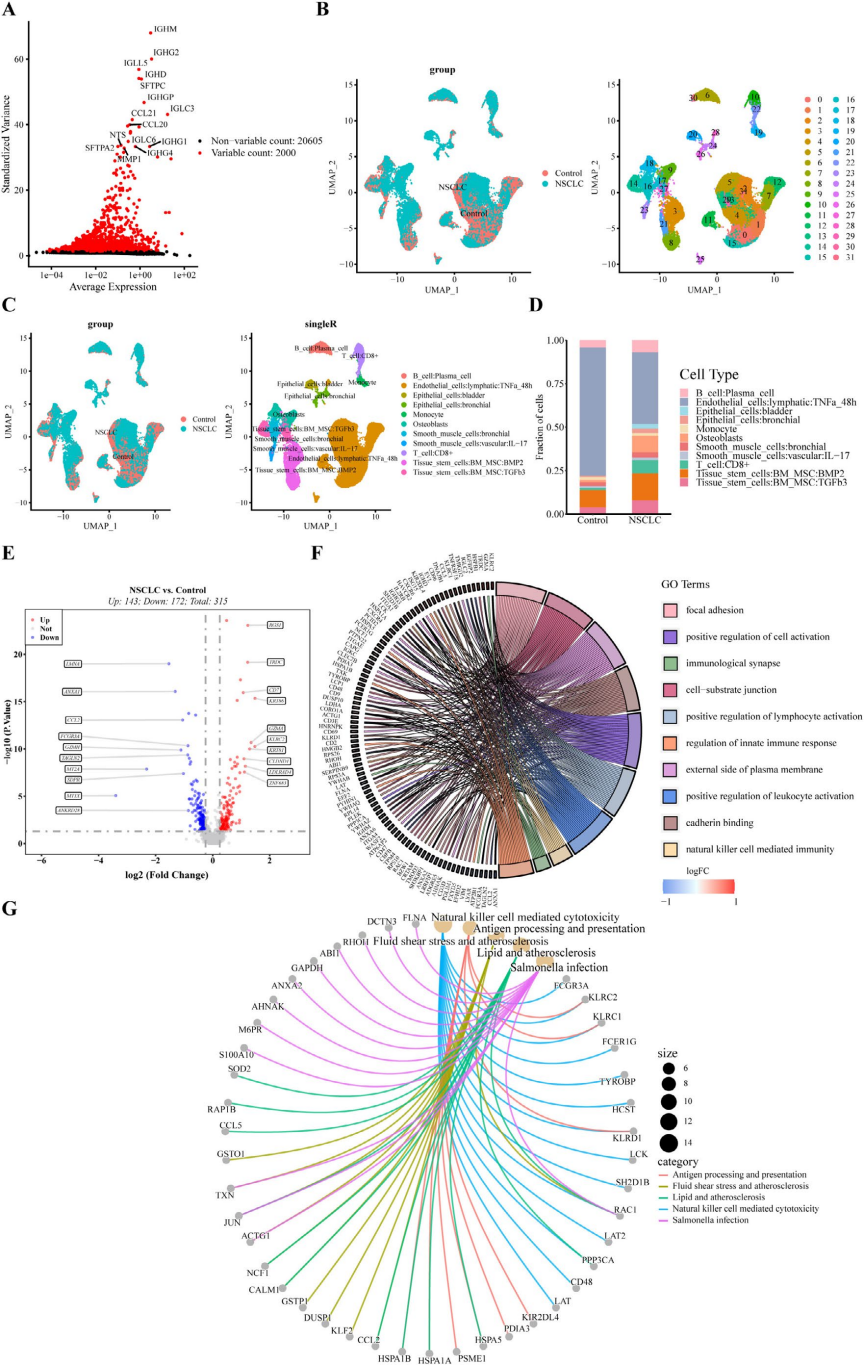
Single‐cell sequencing analysis. (A) Volcano plot of 2000 genes with high variability; the top 20 variable genes are labeled. Red plots represent variable genes, and black plots represent non‐variable genes. (B) Dimension reduction and clustering analysis, resulting in 32 cell clusters. (C) Identification of 8 cell subtypes based on marker genes, including three immune cells (B cell, CD8^+^ T cell, and monocyte) and eight stromal cells. (D) Histogram displaying the proportions of 11 cell subtypes in the NSCLC and control groups. (E) Volcano plot of CD8^+^ T cell‐related DEGs between NSCLC and control groups. (F, G) The top 10 entries of CD8^+^ T cell‐related DEGs by GO analysis shown as a chord diagram, and the top 5 pathways by KEGG analysis displayed as a network diagram.

Single‐cell sequencing data were retrieved from the GSE183219 dataset. After quality control, which confirmed the absence of significant batch effects across samples, genes exhibiting high variability in expression levels between cells were selected. A total of 2000 genes with substantial variation in expression were identified using the Find Variable Features function and the vst method. The top 20 of these genes are shown in Figure 3A. Clustering and dimension reduction revealed 32 distinct cell clusters (Figure 3B). Based on the variable gene expression profiles of each cluster, 11 cell types were identified and classified with marker genes using the SingleR package (Figure 3C). These included eight stromal cell types and three immune cell types, with endothelial cells being the most abundant cellular type among the identified clusters. Additionally, compared to control samples, NSCLC exhibited a higher concentration of CD8^+^ T cells (Figure 3D), which aligned with the results of KM survival analysis from the TCGA‐NSCLC dataset and underscored the critical role of CD8^+^ T cells in the immune defense against NSCLC.

Using the Wilcoxon test, four cell types—endothelial cells, epithelial cells, CD8^+^ T cells, and osteoblasts—were found to differ significantly between the NSCLC and control groups (Figure S1). CD8^+^ T cells were selected for further differential gene expression analysis using the Find Markers function on the single‐cell dataset. In the GSE183219 cohort, 315 DEGs were identified (143 upregulated and 172 downregulated) that were closely associated with CD8^+^ T cells (Table S2, Figure 3E). These DEGs were involved in immune‐related processes, such as the positive activation of lymphocytes, as shown by GO and KEGG analyses (Figure 3F,G).

### 3.4 Construction of the CD8 ^+^ T Cell‐Related Gene Model Based on the Risk Scores

**FIGURE 4:**
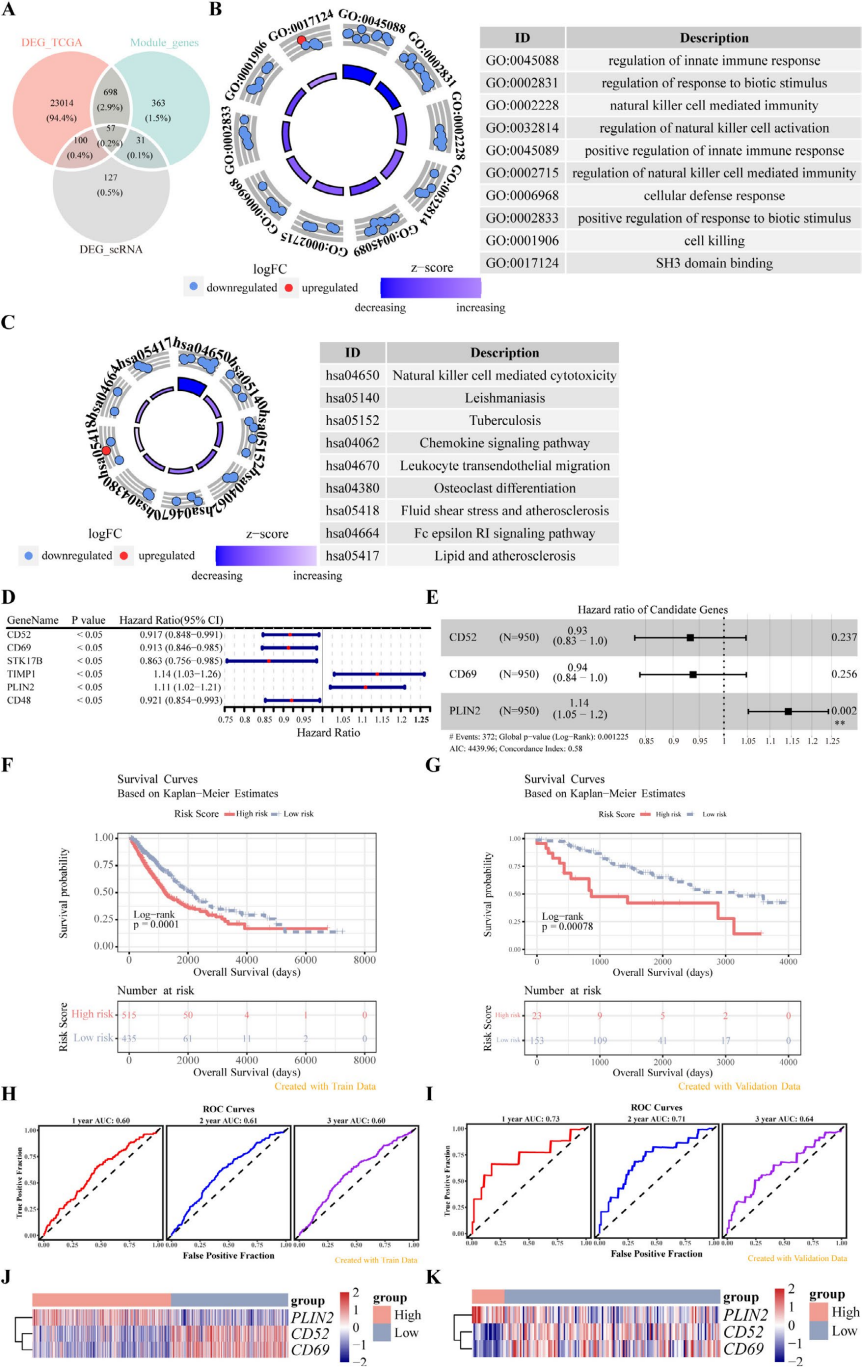
Determination and verification of the prognostic risk model. (A) Venn diagram illustrating the overlapping genes among DEGs, module genes, DEGs of CD8^+^ T cells, and NSCLC. (B) GO annotation for the 57 candidate genes, with the top 10 entries presented in a Bagua map. (C) KEGG analysis of the 57 candidate genes, showing nine enriched pathways in a Bagua map, with corresponding pathways listed. (D) Forest plot displaying six candidate genes identified by univariate Cox regression analysis. (E) Forest plot of three biomarkers associated with CD8^+^ T cells. (F, G) Kaplan–Meier curves for low and high risk score populations from the TCGA‐NSCLC and GSE42127 datasets. (H, I) ROC curves for 1‐, 2‐, and 3‐year survival in the TCGA‐NSCLC and GSE42127 datasets. (J, K) Heatmaps of biomarker expression between the two risk categories in TCGA‐NSCLC and GSE42127 datasets.

To identify more accurate CD8^+^ T cell‐related biomarkers, fifty‐seven candidate genes were identified from the intersection of module genes, DEGs associated with CD8^+^ T cells, and NSCLC. This set included one upregulated gene and 56 downregulated genes (NSCLC vs. control) (Figure 4A). The functional roles of these candidate genes were analyzed through GO and KEGG analyses, visualized as Bagua maps (Figure 4B,C). GO enrichment primarily focused on NK cell‐related immune processes and the regulation of innate immune responses (Figure 4B). KEGG analysis revealed pathways such as NK cell‐mediated cytotoxicity, chemokine signaling, and leukocyte transendothelial migration (Figure 4C), with results predominantly related to immune functions and NSCLC.

Univariate Cox regression analysis identified six out of the 57 candidate genes as significantly associated with clinical outcomes in patients with NSCLC (*p* < 0.05) (Figure 4D). Following validation by LASSO and multivariate Cox regression analysis (Figure S2A,B, Figure 4E), three genes (*CD52*, *CD69*, and *PLIN2*) were confirmed as CD8^+^ T cell‐related biomarkers. A novel predictive risk model was developed using these genes, with the following formula: risk score = (−0.118) × normalized expression of *CD52* + (−0.039) × normalized expression of *CD69* + (0.099) × normalized expression of *PLIN2*. After calculating risk scores for both the TCGA‐NSCLC and GSE42127 datasets, optimal thresholds for risk scores were determined (0.2468788 for TCGA‐NSCLC and 0.1974769 for GSE42127), categorizing patients with NSCLC into high‐and low‐risk groups.

KM survival analysis revealed that patients in the low‐risk group had a longer OS compared to those in the high‐risk group (Figure 4F,G). ROC analysis provided area under the curve (AUC) values of 0.60, 0.61, and 0.60 for 1‐, 2‐, and 3‐year survival in the TCGA‐NSCLC cohort, and 0.73, 0.71, and 0.64 in the GSE42127 dataset, respectively, indicating favorable predictive accuracy of the risk model (Figure 4H,I). Moreover, *PLIN2* was upregulated, while *CD52* and *CD69* were downregulated in high‐risk samples (Figure 4J,K). Consistent findings across the training and validation datasets validated the robustness of the risk model.

### 3.5 Establishment and Verification of a Clinical Prognostic Model

**FIGURE 5:**
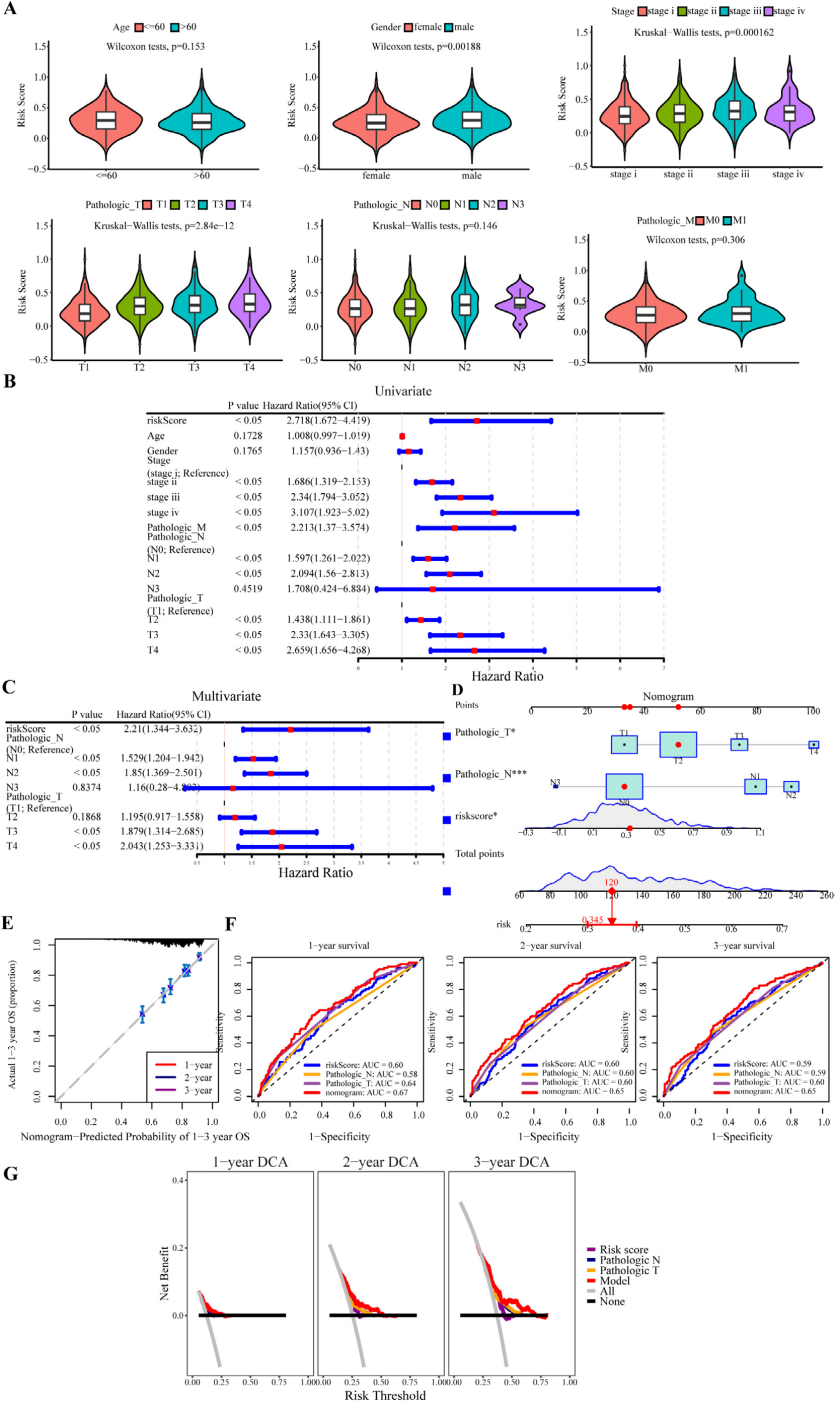
Development and evaluation of the nomogram. (A) Violin plots illustrating the distribution of risk scores across clinical characteristics. Kruskal‐Wallis and Wilcoxon tests showed significant differences in risk scores based on age, stage, and pathological T stage (*p* < 0.05). (B) Univariate Cox regression analysis to identify prognosis‐related factors. Risk score, stage, and pathological TNM stage showed a strong association with survival rate (*p* < 0.05). (C) Multivariate Cox regression analysis identified independent predictors: Risk score, pathological N stage, and T stage (*p* < 0.05). (D) Nomogram combining pathological N and T stages with risk score for prognosis prediction. (E) Calibration plot showing excellent predictive capability of the nomogram. (F) ROC curves for the nomogram and other prognostic indicators at 1‐, 2‐, and 3‐year survival outcomes. The nomogram demonstrated higher accuracy than other indicators across all time points (1‐year AUC = 0.67 vs. 0.60/0.58/0.64, 2‐year AUC = 0.65 vs. 0.60/0.60/0.60, 3‐year AUC = 0.65 vs. 0.59/0.59/0.60). (G) Decision curve analysis showing the superior benefit of the nomogram compared to all other indicators. *P < 0.05, **P < 0.01, ***P < 0.001, and ns no significant difference.

To develop a reliable prognostic prediction model, a nomogram was constructed by integrating clinical indicators and risk scores. First, risk scores were assigned based on various clinical characteristics. Male patients, those with advanced tumor stages, and higher pathological T stages were associated with higher risk scores (Figure 5A). Although the pathological N stage did not show statistical significance (*p* = 0.146), the risk scores for N2 and N3 stages were notably higher than those for N0 and N1 stages. Next, univariate Cox regression analysis was used to evaluate the impact of the risk score, stage, and pathological M, N, and T stages on OS in patients with NSCLC (*p* < 0.05, Figure 5B). Multivariate Cox regression analysis identified the risk score and pathological N and T stages as independent prognostic factors for nomogram construction (Figure 5C,D). Calibration curves confirmed that the 1‐, 2‐, and 3‐year survival curves closely aligned with the ideal curve (Figure 5E), demonstrating excellent fit and accuracy. The nomogram for survival prediction exhibited the highest AUC values—0.67 at 1 year, 0.65 at 2 years, and 0.65 at 3 years—when compared to other indicators in ROC curve analysis (Figure 5F). DCA showed that the clinical prognostic model offered greater predictive benefit for NSCLC mortality compared to individual indicators (Figure 5G). These results suggest that the nomogram can effectively predict the prognosis of patients with NSCLC.

### 3.6 GSEA Analysis Reveals Differential Pathway Activation in High Versus Low‐Risk Populations

**FIGURE 6:**
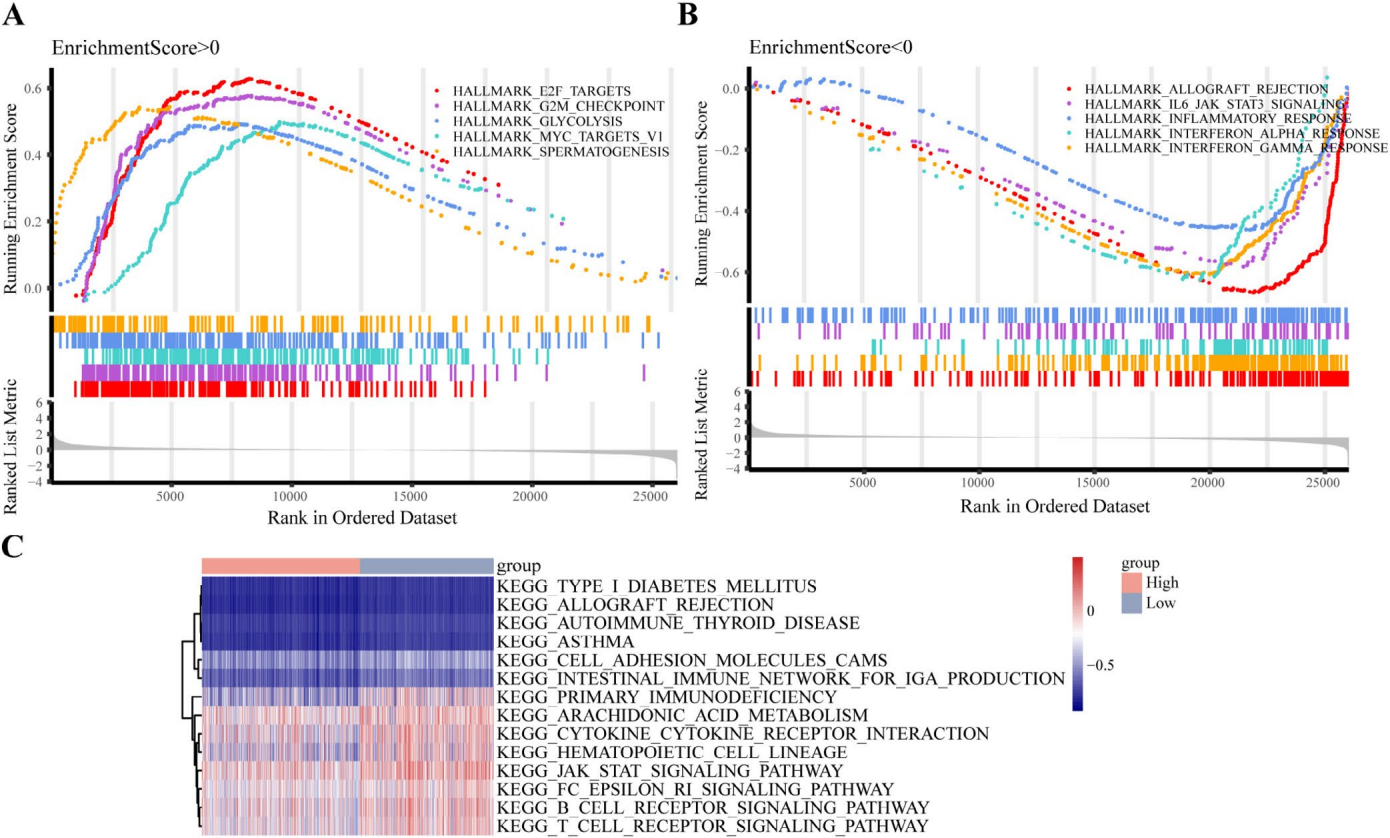
GSEA and ssGSEA of high‐risk and low‐risk cohorts from the TCGA‐NSCLC dataset. (A, B) Bar code diagrams depicting the top 5 pathways with positive and negative enrichment scores, respectively. (C) Heatmap of the top 15 KEGG pathways associated with the risk score through ssGSEA.

GSEA using the HALLMARK gene set revealed that genes upregulated in high‐risk populations were linked to pro‐oncogenic pathways, including E2F targets, MYC targets, glycolysis, and cell cycle processes like the G2M checkpoint (Figure 6A). In contrast, upregulated genes in low‐risk samples were enriched in immune‐related pathways, particularly IL6 JAK STAT3 signaling (Figure 6B). As shown in Figure 6C, the high‐risk group had lower ssGSEA scores for 15 KEGG pathways, primarily related to metabolism and immune responses. These results suggest that high‐risk individuals experience immune suppression, whereas low‐risk individuals display a robust immune landscape.

### 3.7 The Immune System Varies in Landscape Between the Two Distinct Risk Categories

**FIGURE 7:**
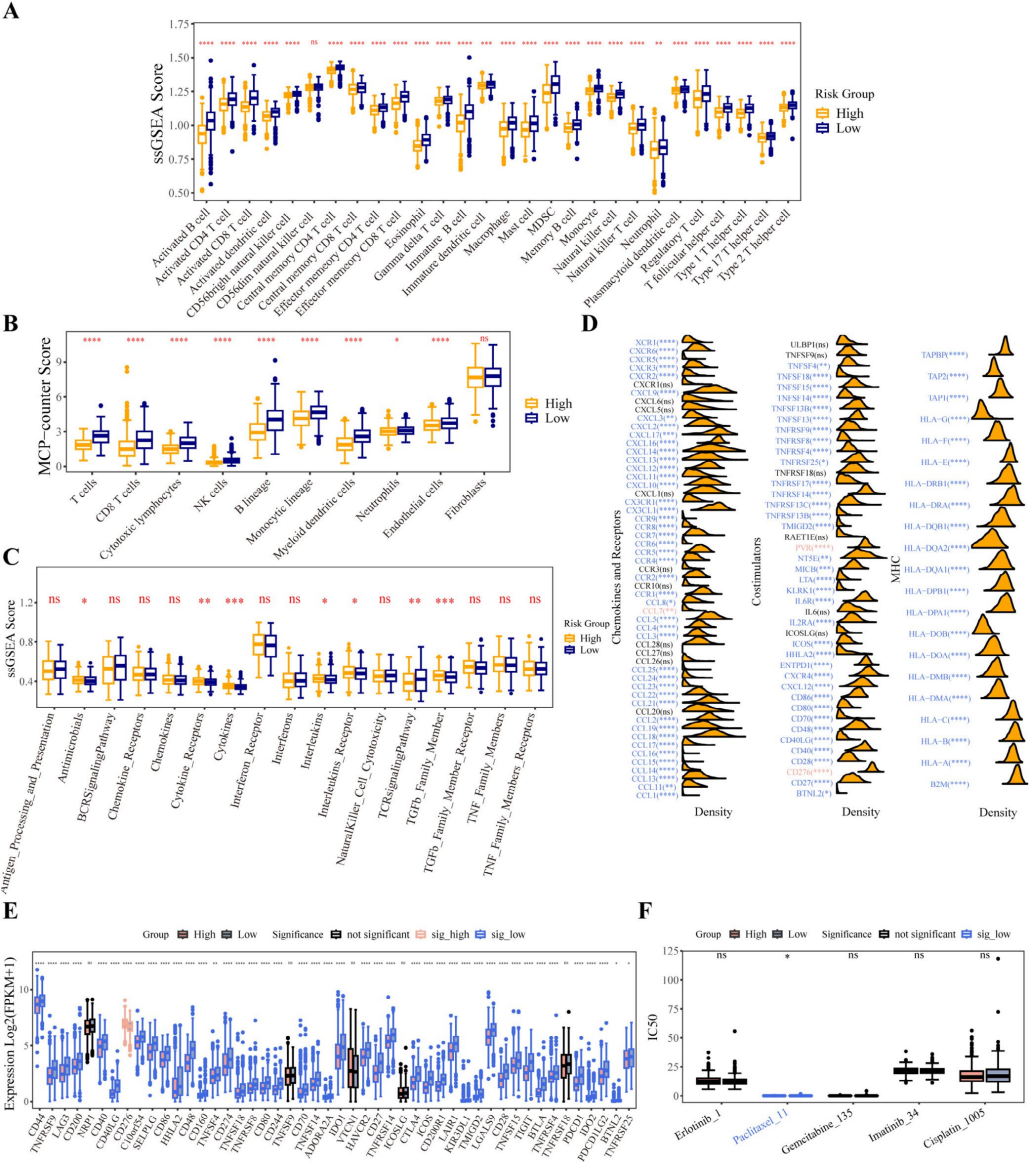
Analysis of the TIME and chemotherapy drug sensitivity. (A) ssGSEA values for each immune cell type in high‐ and low‐risk cohorts. (B) Immune cell infiltration analysis using the MCP‐counter algorithm. (C) ssGSEA values for immune‐related pathways. Seven pathways showed significant differences between the high‐ and low‐risk groups (*p* < 0.05). (D) Differentially expressed chemokines, receptors, co‐stimulators, and MHC molecules in the high‐ and low‐risk cohorts. (E) Differences in immune checkpoint expression levels between the high‐risk and low‐risk cohorts. (F) Sensitivity analysis of five primary chemotherapeutics. ^ns^ *p* > 0.05, *p < 0.05, **p < 0.01, ***p < 0.001, ****p < 0.000.

In the TCGA NSCLC dataset, we performed multi‐dimensional analyses of immune‐related indicators, including immune cells, immune pathways, and active substances. The ssGSEA algorithm was used to identify the abundance values of 28 immune cell types and 17 immune‐related pathways, revealing significant differences in immune system infiltration patterns and activation characteristics between the two groups. The scores of the 28 immune cell types varied greatly between the high‐risk and low‐risk groups, with enrichment scores being lower in the high‐risk group compared to the low‐risk group. Differential analysis showed that, except for CD56dim natural killer cells, 27 immune cell types (including Activated B cells, Neutrophils, and Memory B cells) exhibited significant differences in scores between the high‐risk and low‐risk groups (*p* < 0.01), and the immune scores of these 27 cell types were significantly reduced in the high‐risk group (Figure 7A). The high infiltration of these cells may inhibit tumor progression by enhancing tumor cell clearance efficiency. Furthermore, validation via the MCP‐counter algorithm confirmed that 9 immune cell types, including T cells, CD8^+^ T cells, Cytotoxic lymphocytes, NK cells, B lineage, Monocytic lineage, Myeloid dendritic cells, Neutrophils, and Endothelial cells, were also significantly downregulated in the high‐risk group (Figure 7B). In addition, the abundance values of immune‐related pathways differed significantly between the two groups: the TCR signaling pathway was upregulated in the low‐risk group, while scores for TGF‐β family members, cytokines, interleukins, and their receptors were higher in the high‐risk group (Figure 7C). This indicates that the TME in high‐risk patients has skewed toward immunosuppression, creating conditions for tumors to escape immune surveillance. Moreover, the antimicrobial pathway was also upregulated in the high‐risk group. These findings indicate that the risk score can effectively reflect the functional status of the immune system.

CYT, an important indicator of the anti‐tumor immune activity of CD8^+^ cytotoxic T cells, is determined by the expression levels of PRF1 and GZMA. Consistent with the immune scores of immune cells, the CYT level, as well as the levels of PRF1 and GZMA, was lower in the high‐risk group (Figure S3A–C), suggesting higher CD8^+^ T cell activity in low‐risk individuals. Meanwhile, in the low‐risk group, 35 out of 43 co‐stimulatory factors showed significant differential expression, with PVR and CD276 being upregulated, along with the remaining 33 co‐stimulatory factors, 21 MHC molecules, and 44 chemokines and their receptors (Figure 7D). Notably, among 48 immune checkpoints, 5 (including NRP1) showed no significant difference in expression between the two groups; only CD276 was upregulated in the high‐risk group, while 42 immune checkpoints (including CD44) were more highly expressed in the low‐risk group (Figure 7E). The overexpression of immune checkpoints is closely associated with immune evasion in the disease. Beyond immune‐related analyses, drug sensitivity analysis was performed to identify effective chemotherapeutic agents for each group. As shown in Figure 7F, patients in the high‐risk group showed a better response to paclitaxel, as evidenced by a significantly lower IC50 in this group. These differences provide a clear biological basis for the prognostic value of the risk model.

## 4 Discussion

CD8^+^ T cells, key effector cells in initiating the adaptive immune response, play a critical role in antitumor immunotherapy. In this study, three CD8^+^ T cell‐related genes—*CD52*, *CD69*, and *PLIN2*—were identified as potential biomarkers for NSCLC, marking the first time these genes have been associated with the disease. A prognostic risk model was developed using these biomarkers, and results from both training and validation datasets confirmed that the model could reflect the survival rate of patients with NSCLC to some extent. To enhance the reliability of the prognostic model, clinical features were integrated, leading to the creation of a nomogram. This nomogram outperformed single indicators in predicting clinical outcomes, with accuracy improvements of approximately 5% to 7% after multiple rounds of verification (*p* < 0.001, C‐index 0.66 vs. 0.5967/0.59/0.6133).

CD8^+^ T cells were the core effector cells of anti‐tumor, whose antitumor effects were affected by the functional states and spatial distribution. Long‐term exposure to chronic inflammatory infiltration and tumor intrinsic metabolic reprogramming compelled CD8^+^ T cell exhaustion. Among these three biomarkers, *CD52* as a myeloid activation marker is associated with the activation of immune cells and the recruitment of lymphocytes. *CD52* has been revealed to inhibit the Toll‐like receptor activation of the NF‐κB signaling pathway to deplete inflammation that assists in recovering the functional states of CD8^+^ T cells. *CD69* as the early activation marker of T cells, generally participates in the residence of lymphocytes in lymph nodes and tissues [41], which integrates with S1P1 and favors T cell complete activation, eventually modulating their spatial distribution. For the regulation of metabolism, *CD69* also enhances the stability and distribution of LAT1‐CD98, modulating the tryptophan uptake to better fulfill metabolic demands for an effective immunological response [42]. Lipid droplet‐related *PLIN2‐*mediated abnormal metabolism characterized by the lipid accumulation [43] which is one of the causes of inducing CD8^+^ T cell exhaustion [44]. Meanwhile, *PLIN2* significantly affects the generation and breakdown of lipid droplets, which provide energy for tumor cells through ketone metabolism. It primarily accumulates on CD68^+^ tumor‐associated macrophages within the TIME, leading to a dysregulated immune balance and impaired activation of CD8^+^ T cells [45]. Elevated *PLIN2* levels correlate with shorter OS, higher TNM stage, and poorer prognosis in patients with oral squamous cell carcinoma [45]. Thus, *CD52*, *CD69*, and *PLIN2* orchestrate a tripartite influence on CD8^+^ T cell potency—governing their functional state, spatial positioning, and metabolic capacity—which together underpin the prognostic power of our risk model.

The immune microenvironment in individuals with NSCLC is closely linked to heterogeneous clinical responses to treatment. Based on the expression of three key biomarkers, the prognostic risk model stratified patients with NSCLC into high‐ and low‐risk categories. Patients in the high‐risk group exhibited poorer OS. The functions enriched in the high‐risk cohort were primarily associated with the cell cycle and tumor progression. Disruption in cell cycle regulation leads to uncontrolled cell proliferation, driving malignant progression. GSEA analysis further revealed that the high‐risk group had lower enrichment in immune‐related pathways compared to the low‐risk group, which showed greater enrichment in immune response and metabolism. These findings suggest that the clinical outcomes of patients with NSCLC are influenced by the extent of immune infiltration, which can be predicted by CD8^+^ T cell‐related genes.

The interaction between immune cells and tumor cells within the tumor microenvironment (TME) plays a critical role in determining the anti‐tumor immune response, a fundamental mechanism underlying immunotherapy. Immune checkpoint inhibitors (ICIs) targeting CTLA‐4, PD‐1, and PD‐L1 have been the most effective immunotherapeutic strategies for NSCLC. However, a significant proportion of patients still fail to respond to these treatments. Given the importance of the TME in clinical immunotherapy, the immune landscape between the high‐ and low‐risk subgroups was further evaluated. The abundance of immune cells and the expression of co‐stimulators, MHCs, chemokines, and receptors were significantly higher in the low‐risk group. CD8^+^ T cells and NK cells, key effectors of anti‐tumor immunity, were more abundant in this cohort. B cells can act as APCs to activate T cell‐mediated anti‐tumor responses [46]. Chemokines such as CXCL4, CXCL9, CXCL10, CXCL11, CXCL13, and CCL5 contribute to the accumulation of CD8^+^ T cells and B lymphocytes in tumor tissues and inhibit angiogenesis [47, 48, 49]. MHC I and II molecules present antigenic peptides on the cell surface, allowing CD8^+^ T cells to recognize and target tumor cells expressing aberrant proteins [50]. Analysis of immune‐related pathways revealed that the TCR signaling pathway exhibited a higher immunological score in the low‐risk group. TCR signaling plays a critical role in identifying and eliminating cancer cells and is essential for the differentiation of naïve T cells into CD8^+^ T cells. The current research analyzed that tumor vaccines designed based on TCR‐engineered T cells offer benefits compared to chimeric antigen receptor (CAR)‐T cell vaccines [51]. These implicate that low‐risk patients better respond to immunotherapies and exert stronger anti‐tumor abilities.

TGF‐β family members, interleukins, cytokines, and their receptors were more prominent in the high‐risk group. The interleukin family, consisting of several key cytokines, plays a significant role in tumor‐promoting activities. For instance, TGF‐β is known to impair anti‐tumor immunity and contribute to tumor escape from immune surveillance, suggesting that inhibiting TGF‐β could enhance the immunotherapeutic effects of CD8^+^ T cells and NK cells [52]. IL‐10, secreted by tumor‐associated macrophages, stimulates cancer stem cell‐like characteristics in NSCLC cells via the JAK1/STAT1/NF‐κB/Notch1 pathway [53]. Additionally, IL‐6 and IL‐8 promote angiogenesis in NSCLC by inducing VEGF expression [54, 55], while long‐term exposure to IL‐1β induces a memory‐like epithelial–mesenchymal transition (EMT) phenotype in lung cancer tissues [54]. These findings indicate a “cold” TME in the high‐risk group, characterized by immune suppression, which likely reduces sensitivity to immunotherapy.

The high‐risk group also displayed decreased expression of immune checkpoints, with CD276 being the only upregulated checkpoint. This suggests potential new avenues for immunotherapy in high‐risk patients with NSCLC. CD276 is widely expressed in NSCLC tissues and functions as both a co‐stimulator and co‐inhibitor. Current studies show that CD276 inhibits T‐cell proliferation, reducing IL‐2 and IFN‐γ production [56], and induces CD8^+^ T cell exhaustion. The upregulation of CD276 expression in the high‐risk group, as noted in immune checkpoint analysis, suggests that CD276 may be a promising target for ICIs in this cohort. Present studies are focusing on the emerging anti‐tumor strategy that targets CD276 through CAR‐T cells [57], but the clinical proof of this therapeutic method has not been completed yet. Our risk model can be used as a “stratification factor” for the clinical trial of this project that enrolls CD276‐upregulated patients to effectively avoid overall response rate dilution due to the heterogeneity of enrolled patients.

The function of antimicrobial pathways was also upregulated in the high‐risk group. Notably, CCL7 was highly expressed in these patients. CCL7 is known to promote a robust protective response against *Cryptococcus neoformans* infection in mice [58]. Pulmonary infections are common complications in advanced lung cancer and can be fatal. *Cryptococcus* is a frequent pathogen that can cause pulmonary infections in individuals with immune suppression [59]. Thus, patients in the high‐risk cohort are more susceptible to pulmonary infections, suggesting that adjuvant antibacterial therapy, in conjunction with cancer treatment, should be considered. In the chemotherapy drug sensitivity analysis, paclitaxel, a standard first‐line treatment for NSCLC, exhibited a significantly lower IC50 in the high‐risk group. Paclitaxel binds to microtubulin, inhibiting its dissociation, which disrupts cell cycle progression and promotes the release of tumor cell antigens [60]. These findings align with GSEA results, which showed that cell cycle‐related pathways were enriched in the high‐risk group.

Beyond insights into immune cells and drug sensitivity, this risk model holds substantial clinical significance for the personalized management of NSCLC. First, it enables prognostic stratification relative to traditional detection methods. This addresses the limitations of conventional staging in capturing immune heterogeneity, thereby optimizing risk‐adapted strategies. Second, by linking immune phenotypes to therapeutic responses, the model guides treatment selection. Low‐risk patients, characterized by active antitumor immune responses, are more likely to respond to ICIs targeting PD‐1/PD‐L1. For clinical translation, risk scores could be used to prioritize ICIs as first‐line therapy for this subgroup, reducing unnecessary toxicity associated with combined chemo‐immunotherapy. Furthermore, the model has the potential for dynamic monitoring of treatment responses. Since its core genes reflect CD8^+^ T cell function, changes in risk scores during immunotherapy can serve as a surrogate marker for immune reactivation.

Therefore, targeting cell cycle activity may be a more effective approach for high‐risk patients, while immunotherapy may yield a more favorable response in low‐risk patients. Collectively, our risk model offers a valuable tool for selecting appropriate immunotherapeutic targets and chemotherapeutic agents in patients with NSCLC. Conventional ICIs targeting CTLA‐4, PD‐1, and PD‐L1 are likely more beneficial for low‐risk patients, while ICIs targeting CD276 and the chemotherapeutic agent paclitaxel may be more effective for high‐risk patients with NSCLC.

## 5 Conclusion

This study provides a novel approach to classifying patients with NSCLC and selecting tailored treatment options. However, it has several limitations. First, the study focused exclusively on OS, without considering progression‐free survival or treatment response rates, which limits its applicability for dynamic treatment decisions. Second, the study only assessed five chemotherapy drugs and did not include targeted therapies (such as EGFR‐TKIs) or immunotherapy, limiting its clinical guidance value. Third, the risk model's relatively low AUC values (0.6, 0.6, and 0.59) indicate that its reliability needs further improvement. Lastly, the response to immunotherapy and chemotherapy based on CD276 and paclitaxel requires validation through comprehensive prospective clinical cohort studies.

## Author Contributions

**Yi‐yang Jiang:** writing‐original draft (equal). **Min‐min Yu:** data curation (supporting), formal analysis (supporting), software (supporting). **Xia Cui:** data curation (supporting), formal analysis (supporting), software (supporting). **Xue Li:** data curation (supporting), formal analysis (supporting), methodology (supporting), software (supporting). **Bin‐bin Li:** data curation (supporting), formal analysis (supporting), resources (supporting), software (supporting). **Jing‐tao Zhang:** investigation (supporting), methodology (lead). **Fei Xu:** funding acquisition (lead), supervision (lead), writing – review and editing (lead).

## Ethics Statement

This study did not involve human subjects, live animals, or any interventional procedures requiring ethical review. Research data were sourced from publicly available databases, with all identifying information removed. Data acquisition complied with relevant data‐sharing protocols. Such research does not require ethical review approval; therefore, no ethics committee approval was obtained, and no informed consent from subjects was involved.

## Consent

All authors confirm their consent for publication of the manuscript.

## Conflicts of Interest

The authors declare no conflicts of interest.

## Supporting information


**Figure S1:** Comparison of the proportion of different cells in the NSCLC and normal cohort. There are notable differences in the proportions of endothelial cells, epithelial cells, osteoblasts, and CD8^+^ T cells between the NSCLC and normal cohorts.

**Figure S2:** Selection of feature genes based on LASSO regression analysis. (A) The optimal value of the parameter lambda was determined using tenfold cross‐validation. The lambda value at the left dotted line represents the minimum model deviation, while the lambda value at the right dotted line represents one standard deviation of the minimum model deviation. Values above the plot indicate the number of filtered feature genes. (B) Diagram showing the relationship between lambda and regression coefficients. Each gene is represented by a curve, and the lambda value corresponding to the optimal model performance is indicated by the dotted line. Five of the six genes were selected for subsequent analysis.

**Figure S3:** Differential analysis of CYT scores. Elevated expression of *PRF1* (A) and *GZMA* (B), along with a higher total CYT score (C), was observed in the low‐risk population.

**Table S1:** Differentially expressed genes between the NSCLC and normal cohorts.

**Table S2:** Genes associated with CD8^+^ T cells that differ between the NSCLC and normal populations.

## Data Availability

Data will be made available on request.

## References

[1] F. Bray , M. Laversanne , H. Sung , et al., “Global Cancer Statistics 2022: GLOBOCAN Estimates of Incidence and Mortality Worldwide for 36 Cancers in 185 Countries,” CA: A Cancer Journal for Clinicians 74, no. 3 (2024): 229–263. 38572751 10.3322/caac.21834

[2] A. K. Ganti , A. B. Klein , I. Cotarla , B. Seal , and E. Chou , “Update of Incidence, Prevalence, Survival, and Initial Treatment in Patients With Non‐Small Cell Lung Cancer in the US,” JAMA Oncology 7, no. 12 (2021): 1824–1832. 34673888 10.1001/jamaoncol.2021.4932 PMC8532041

[3] C. Wang , Q. Zeng , Z. M. Gül , et al., “Circadian Tumor Infiltration and Function of CD8+ T Cells Dictate Immunotherapy Efficacy,” Cell 187, no. 11 (2024): 2690–2702.e17. 38723627 10.1016/j.cell.2024.04.015

[4] Z. Bai , X. Cheng , T. Ma , et al., “CD8+ T Cells Infiltrating Into Tumors Were Controlled by Immune Status of Pulmonary Lymph Nodes and Correlated With Non‐Small Cell Lung Cancer (NSCLC) Patients' Prognosis Treated With Chemoimmunotherapy,” Lung Cancer 197 (2024): 107991. 39454350 10.1016/j.lungcan.2024.107991

[5] Q. Yan , S. Li , L. He , and N. Chen , “Prognostic Implications of Tumor‐Infiltrating Lymphocytes in Non‐Small Cell Lung Cancer: A Systematic Review and Meta‐Analysis,” Frontiers in Immunology 15 (2024): 1476365. 39372398 10.3389/fimmu.2024.1476365 PMC11449740

[6] L. Yang , H. Yang , M. Zhao , et al., “Stage‐Dependent Spatial Distribution and Prognostic Value of CD8(+) Tissue‐Resident Memory T Cells in NSCLC,” npj Precision Oncology 9, no. 1 (2025): 51. 39987153 10.1038/s41698-025-00831-x PMC11846915

[7] G. Yang , S. Cai , M. Hu , et al., “Functional Status and Spatial Architecture of Tumor‐Infiltrating CD8+ T Cells Are Associated With Lymph Node Metastases in Non‐Small Cell Lung Cancer,” Journal of Translational Medicine 21, no. 1 (2023): 320. 37173705 10.1186/s12967-023-04154-y PMC10182600

[8] H. Huang , X. Zhu , Y. Yu , et al., “EGFR Mutations Induce the Suppression of CD8(+) T Cell and Anti‐PD‐1 Resistance via ERK1/2‐p90RSK‐TGF‐β Axis in Non‐Small Cell Lung Cancer,” Journal of Translational Medicine 22, no. 1 (2024): 653. 39004699 10.1186/s12967-024-05456-5 PMC11246587

[9] L. G. Morosi , G. M. Piperno , L. López , et al., “ALCAM‐Mediated cDC1 CD8 T Cells Interactions Are Suppressed in Advanced Lung Tumors,” Oncoimmunology 13, no. 1 (2024): 2367843. 38887373 10.1080/2162402X.2024.2367843 PMC11181928

[10] R. E. Wessel , N. Ageeb , J. M. Obeid , et al., “Spatial Colocalization and Combined Survival Benefit of Natural Killer and CD8 T Cells Despite Profound MHC Class I Loss in Non‐Small Cell Lung Cancer,” Journal for Immunotherapy of Cancer 12, no. 9 (2024): e009126. 39299754 10.1136/jitc-2024-009126 PMC11418484

[11] S. Mestiri , A. Sami , N. Sah , et al., “Cellular Plasticity and Non‐Small Cell Lung Cancer: Role of T and NK Cell Immune Evasion and Acquisition of Resistance to Immunotherapies,” Cancer Metastasis Reviews 44, no. 1 (2025): 27. 39856479 10.1007/s10555-025-10244-8

[12] B. Zhang , J. Liu , Y. Mo , K. Zhang , B. Huang , and D. Shang , “CD8+ T Cell Exhaustion and Its Regulatory Mechanisms in the Tumor Microenvironment: Key to the Success of Immunotherapy,” Frontiers in Immunology 15 (2024): 1476904. 39372416 10.3389/fimmu.2024.1476904 PMC11452849

[13] F. Alfei , K. Kanev , M. Hofmann , et al., “TOX Reinforces the Phenotype and Longevity of Exhausted T Cells in Chronic Viral Infection,” Nature 571, no. 7764 (2019): 265–269. 31207605 10.1038/s41586-019-1326-9

[14] C. Yao , H. W. Sun , N. E. Lacey , et al., “Single‐Cell RNA‐Seq Reveals TOX as a Key Regulator of CD8(+) T Cell Persistence in Chronic Infection,” Nature Immunology 20, no. 7 (2019): 890–901. 31209400 10.1038/s41590-019-0403-4 PMC6588409

[15] K. Sakuishi , S. F. Ngiow , J. M. Sullivan , et al., “TIM3(+)FOXP3(+) Regulatory T Cells Are Tissue‐Specific Promoters of T‐Cell Dysfunction in Cancer,” Oncoimmunology 2, no. 4 (2013): e23849. 23734331 10.4161/onci.23849 PMC3654601

[16] L. P. Andrews , S. C. Butler , J. Cui , et al., “LAG‐3 and PD‐1 Synergize on CD8(+) T Cells to Drive T Cell Exhaustion and Hinder Autocrine IFN‐γ‐Dependent Anti‐Tumor Immunity,” Cell 187, no. 16 (2024): 4355–4372.e22. 39121848 10.1016/j.cell.2024.07.016 PMC11323044

[17] B. Allard , D. Allard , L. Buisseret , and J. Stagg , “The Adenosine Pathway in Immuno‐Oncology,” Nature Reviews Clinical Oncology 17, no. 10 (2020): 611–629. 10.1038/s41571-020-0382-2 32514148

[18] N. Kaplinsky , K. Williams , D. Watkins , M. Adams , L. Stanbery , and J. Nemunaitis , “Regulatory Role of CD39 and CD73 in Tumor Immunity,” Future Oncology 20, no. 19 (2024): 1367–1380. 38652041 10.2217/fon-2023-0871 PMC11321403

[19] D. S. Thommen , V. H. Koelzer , P. Herzig , et al., “A Transcriptionally and Functionally Distinct PD‐1(+) CD8(+) T Cell Pool With Predictive Potential in Non‐Small‐Cell Lung Cancer Treated With PD‐1 Blockade,” Nature Medicine 24, no. 7 (2018): 994–1004. 10.1038/s41591-018-0057-z PMC6110381 29892065

[20] J. Zeng , L. Zhang , S. Ma , et al., “Dysregulation of Peripheral and Intratumoral KLRG1(+) CD8(+)T Cells Is Associated With Immune Evasion in Patients With Non‐Small‐Cell Lung Cancer,” Translational Oncology 45 (2024): 101968. 38713923 10.1016/j.tranon.2024.101968 PMC11097332

[21] S. Davis and P. S. Meltzer , “GEOquery: A Bridge Between the Gene Expression Omnibus (GEO) and BioConductor,” Bioinformatics 23, no. 14 (2007): 1846–1847. 17496320 10.1093/bioinformatics/btm254

[22] S. K. Hight , A. Mootz , R. K. Kollipara , et al., “An In Vivo Functional Genomics Screen of Nuclear Receptors and Their Co‐Regulators Identifies FOXA1 as an Essential Gene in Lung Tumorigenesis,” Neoplasia 22, no. 8 (2020): 294–310. 32512502 10.1016/j.neo.2020.04.005 PMC7281309

[23] J. A. Grout , P. Sirven , A. M. Leader , et al., “Spatial Positioning and Matrix Programs of Cancer‐Associated Fibroblasts Promote T‐Cell Exclusion in Human Lung Tumors,” Cancer Discovery 12, no. 11 (2022): 2606–2625. 36027053 10.1158/2159-8290.CD-21-1714 PMC9633420

[24] M. I. Love , W. Huber , and S. Anders , “Moderated Estimation of Fold Change and Dispersion for RNA‐Seq Data With DESeq2,” Genome Biology 15, no. 12 (2014): 550. 25516281 10.1186/s13059-014-0550-8 PMC4302049

[25] C. Ginestet , “ggplot2: Elegant Graphics for Data Analysis,” Journal of the Royal Statistical Society: Series A (Statistics in Society) 174, no. 1 (2011): 245–246.

[26] Z. Gu , ComplexHeatmap Complete Reference (2022), https://github.com/jokergoo/ComplexHeatmap.

[27] D. Aran , Z. Hu , and A. J. Butte , “xCell: Digitally Portraying the Tissue Cellular Heterogeneity Landscape,” Genome Biology 18, no. 1 (2017): 220. 29141660 10.1186/s13059-017-1349-1 PMC5688663

[28] T. M. Therneau and P. M. Grambsch , “survival: Survival Analysis. R package version 3.3‐3,” https://cran.r‐project.org/package=survival%20.

[29] A. Kassambara , M. Kosinski , and P. Biecek , “survminer: Drawing Survival Curves using 'ggplot2'. R package version 0.4.9,” https://CRAN.R‐project.org/package=survminer.

[30] P. Langfelder and S. Horvath , “WGCNA: An R Package for Weighted Correlation Network Analysis,” BMC Bioinformatics 9 (2008): 559. 19114008 10.1186/1471-2105-9-559 PMC2631488

[31] Y. Xie , H.‐A. Kim , D. R. O'Hallaron , M. K. Reiter , and H. Zhang , “Seurat: A Pointillist Approach to Anomaly Detection,” in Recent Advances in Intrusion Detection, vol. 3224 (Carnegie Mellon University, 2004).

[32] D. Aran , A. P. Looney , L. Liu , et al., “Reference‐Based Analysis of Lung Single‐Cell Sequencing Reveals a Transitional Profibrotic Macrophage,” Nature Immunology 20, no. 2 (2019): 163–172. 30643263 10.1038/s41590-018-0276-y PMC6340744

[33] Y. Zhang , L. Yong , Y. Luo , et al., “Enhancement of HIFU Ablation by Sonosensitizer‐Loading Liquid Fluorocarbon Nanoparticles With Pre‐Targeting in a Mouse Model,” Scientific Reports 9, no. 1 (2019): 6982. 31061456 10.1038/s41598-019-43416-y PMC6502828

[34] Y. Hao , S. Hao , E. Andersen‐Nissen , et al., “Integrated Analysis of Multimodal Single‐Cell Data,” Cell 184, no. 13 (2021): 3573–3587.e29. 34062119 10.1016/j.cell.2021.04.048 PMC8238499

[35] J. Friedman , T. Hastie , and R. Tibshirani , “Regularization Paths for Generalized Linear Models via Coordinate Descent,” Journal of Statistical Software 33, no. 1 (2010): 1–22. 20808728 PMC2929880

[36] P. J. Heagerty , T. Lumley , and M. S. Pepe , “Time‐Dependent ROC Curves for Censored Survival Data and a Diagnostic Marker,” Biometrics 56, no. 2 (2000): 337–344, 10.1111/j.0006-341X.2000.00337.x. 10877287

[37] F. E. Harrell, Jr. , “rms: Regression Modeling Strategies. R package version 6.2‐0,” https://CRAN.R‐project.org/package=rms.

[38] T. Wu , E. Hu , S. Xu , et al., “clusterProfiler 4.0: A Universal Enrichment Tool for Interpreting Omics Data,” Innovation 2, no. 3 (2021): 100141. 34557778 10.1016/j.xinn.2021.100141 PMC8454663

[39] S. Hänzelmann , R. Castelo , and J. Guinney , “GSVA: Gene Set Variation Analysis for Microarray and RNA‐Seq Data,” BMC Bioinformatics 14 (2013): 7. 23323831 10.1186/1471-2105-14-7 PMC3618321

[40] D. Maeser , R. F. Gruener , and R. S. Huang , “oncoPredict: An R Package for Predicting in Vivo or Cancer Patient Drug Response and Biomarkers From Cell Line Screening Data,” Briefings in Bioinformatics 22, no. 6 (2021): bbab260. 34260682 10.1093/bib/bbab260 PMC8574972

[41] D. Cibrián and F. Sánchez‐Madrid , “CD69: From Activation Marker to Metabolic Gatekeeper,” European Journal of Immunology 47, no. 6 (2017): 946–953. 28475283 10.1002/eji.201646837 PMC6485631

[42] D. Cibrian , M. L. Saiz , H. de la Fuente , et al., “CD69 Controls the Uptake of L‐Tryptophan Through LAT1‐CD98 and AhR‐Dependent Secretion of IL‐22 in Psoriasis,” Nature Immunology 17, no. 8 (2016): 985–996. 27376471 10.1038/ni.3504 PMC5146640

[43] H. Luo , X. She , Y. Zhang , et al., “PLIN2 Promotes Lipid Accumulation in Ascites‐Associated Macrophages and Ovarian Cancer Progression by HIF1α/SPP1 Signaling,” Advanced Science 12, no. 12 (2025): e2411314. 39921309 10.1002/advs.202411314 PMC11948008

[44] H. Wang , X. Niu , Z. Jin , et al., “Immunotherapy Resistance in Non‐Small Cell Lung Cancer: From Mechanisms to Therapeutic Opportunities,” Journal of Experimental & Clinical Cancer Research 44, no. 1 (2025): 250. 40849659 10.1186/s13046-025-03519-z PMC12374485

[45] Y. He , Y. Dong , X. Zhang , et al., “Lipid Droplet‐Related PLIN2 in CD68(+) Tumor‐Associated Macrophage of Oral Squamous Cell Carcinoma: Implications for Cancer Prognosis and Immunotherapy,” Frontiers in Oncology 12 (2022): 824235. 35372038 10.3389/fonc.2022.824235 PMC8967322

[46] M. H. Zhang , B. L. Scotland , Y. Jiao , et al., “Lipid‐Polymer Hybrid Nanoparticles Utilize B Cells and Dendritic Cells to Elicit Distinct Antigen‐Specific CD4(+) And CD8(+) T Cell Responses,” ACS Applied Bio Materials 7, no. 8 (2024): 4818–4830. 10.1021/acsabm.3c00229 PMC10665545 37219857

[47] S. Spranger , D. Dai , B. Horton , and T. F. Gajewski , “Tumor‐Residing Batf3 Dendritic Cells Are Required for Effector T Cell Trafficking and Adoptive T Cell Therapy,” Cancer Cell 31, no. 5 (2017): 711–723.e4. 28486109 10.1016/j.ccell.2017.04.003 PMC5650691

[48] D. Dangaj , M. Bruand , A. J. Grimm , et al., “Cooperation Between Constitutive and Inducible Chemokines Enables T Cell Engraftment and Immune Attack in Solid Tumors,” Cancer Cell 35, no. 6 (2019): 885–900.e10. 31185212 10.1016/j.ccell.2019.05.004 PMC6961655

[49] S. Ramachandran , A. K. Verma , K. Dev , et al., “Role of Cytokines and Chemokines in NSCLC Immune Navigation and Proliferation,” Oxidative Medicine and Cellular Longevity 2021 (2021): 5563746. 34336101 10.1155/2021/5563746 PMC8313354

[50] K. Dhatchinamoorthy , J. D. Colbert , and K. L. Rock , “Cancer Immune Evasion Through Loss of MHC Class I Antigen Presentation,” Frontiers in Immunology 12 (2021): 636568. 33767702 10.3389/fimmu.2021.636568 PMC7986854

[51] K. Ma , Y. Xu , H. Cheng , K. Tang , J. Ma , and B. Huang , “T Cell‐Based Cancer Immunotherapy: Opportunities and Challenges,” Science Bulletin 70 (2025): 1872–1890. 40221316 10.1016/j.scib.2025.03.054

[52] J. Kment , D. Newsted , S. Young , et al., “Blockade of TGF‐β and PD‐L1 by Bintrafusp Alfa Promotes Survival in Preclinical Ovarian Cancer Models by Promoting T Effector and NK Cell Responses,” British Journal of Cancer 130, no. 12 (2024): 2003–2015. 38622286 10.1038/s41416-024-02677-9 PMC11183086

[53] L. Yang , Y. Dong , Y. Li , et al., “IL‐10 Derived From M2 Macrophage Promotes Cancer Stemness via JAK1/STAT1/NF‐κB/Notch1 Pathway in Non‐Small Cell Lung Cancer,” International Journal of Cancer 145, no. 4 (2019): 1099–1110. 30671927 10.1002/ijc.32151

[54] D. Masuya , C. Huang , D. Liu , et al., “The Intratumoral Expression of Vascular Endothelial Growth Factor and Interleukin‐8 Associated With Angiogenesis in Nonsmall Cell Lung Carcinoma Patients,” Cancer 92, no. 10 (2001): 2628–2638. 11745198 10.1002/1097-0142(20011115)92:10<2628::aid-cncr1616>3.0.co;2-f

[55] H. Dalwadi , K. Krysan , N. Heuze‐Vourc'h , et al., “Cyclooxygenase‐2‐Dependent Activation of Signal Transducer and Activator of Transcription 3 by Interleukin‐6 in Non‐Small Cell Lung Cancer,” Clinical Cancer Research 11, no. 21 (2005): 7674–7682. 16278387 10.1158/1078-0432.CCR-05-1205

[56] H. J. Liu , H. Du , D. Khabibullin , et al., “mTORC1 Upregulates B7‐H3/CD276 to Inhibit Antitumor T Cells and Drive Tumor Immune Evasion,” Nature Communications 14, no. 1 (2023): 1214. 10.1038/s41467-023-36881-7 PMC9984496 36869048

[57] B. Kristmann , N. Werchau , L. Suresh , et al., “Targeting CD276 With Adapter‐CAR T‐Cells Provides a Novel Therapeutic Strategy in Small Cell Lung Cancer and Prevents CD276‐Dependent Fratricide,” Journal of Hematology & Oncology 18, no. 1 (2025): 76. 40722088 10.1186/s13045-025-01729-8 PMC12305915

[58] M. Zhang , W. Yang , P. Wang , et al., “CCL7 Recruits cDC1 to Promote Antitumor Immunity and Facilitate Checkpoint Immunotherapy to Non‐Small Cell Lung Cancer,” Nature Communications 11, no. 1 (2020): 6119. 10.1038/s41467-020-19973-6 PMC7704643 33257678

[59] K. Yamagata , C. Hirano , N. Kanno , et al., “Pulmonary Nodule in a Patient With Oral and Lung Cancer: Cryptococcus Infection,” Dentistry Journal 7, no. 4 (2019): 102. 31652805 10.3390/dj7040102 PMC6961046

[60] Z. Xu , L. Zheng , and S. Li , “Paclitaxel‐Induced Inhibition of NSCLC Invasion and Migration via RBFOX3‐Mediated circIGF1R Biogenesis,” Scientific Reports 14, no. 1 (2024): 774. 38191906 10.1038/s41598-024-51500-1 PMC10774373

