# Supplementary figures and images for: Integration of Transcriptome Profiling and Single‐Cell Sequencing Analysis to Establish a CD8+ T Cell–Related Prognostic Model for Patients With NSCLC: From Assessment to Therapy
Source: Cancer Med. 2025 Nov 12;14(21):e71337. doi: 10.1002/cam4.71337 (PMC12611320; doi:10.1002/cam4.71337)

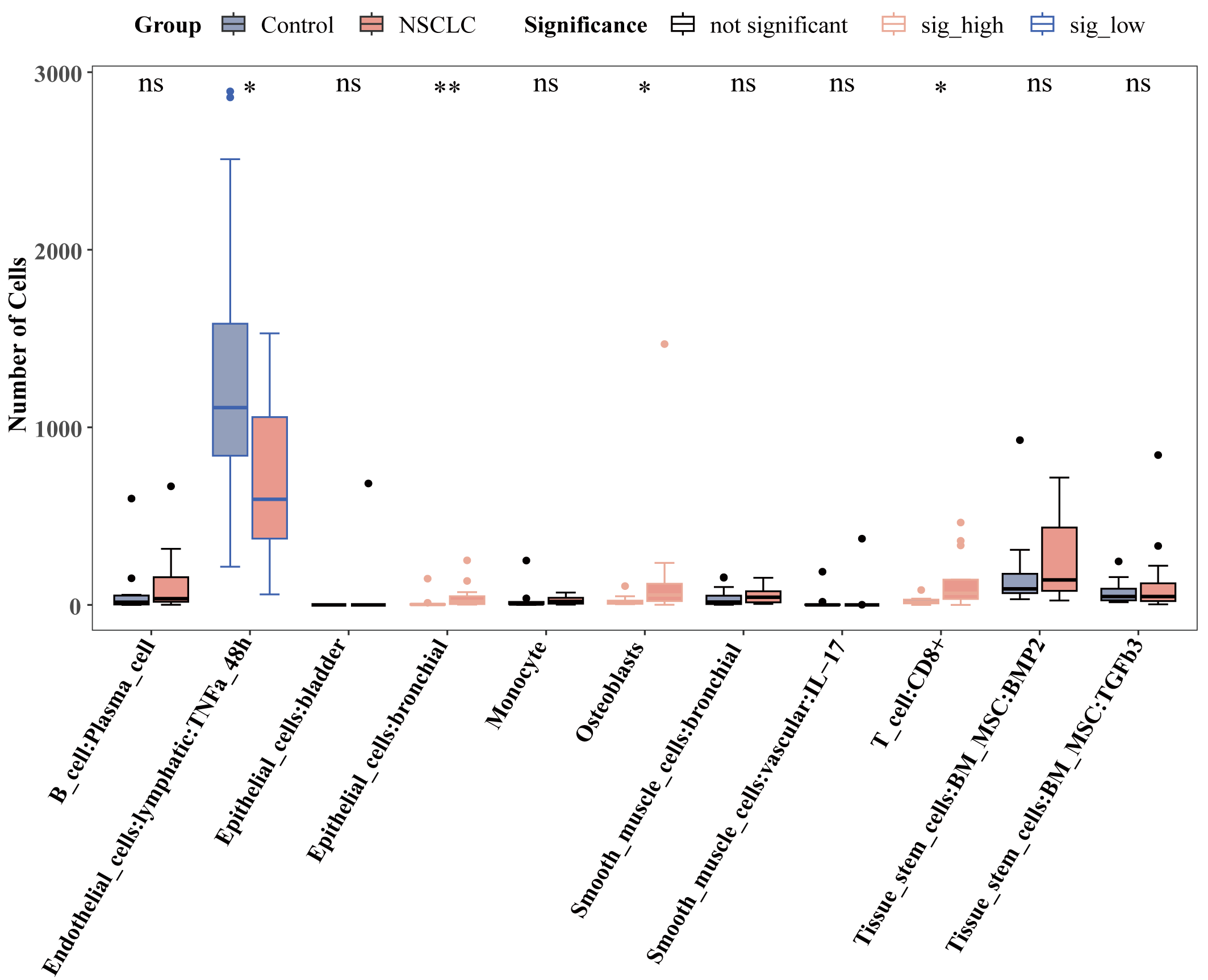

Supplement: Supplementary file 1 — Figure S1: Comparison of the proportion of different cells in the NSCLC and normal cohort. There are notable differences in the proportions of endothelial cells, epithelial cells, osteoblasts, and CD8+ T cells between the NSCLC and normal cohorts. [file CAM4-14-e71337-s002.tif]

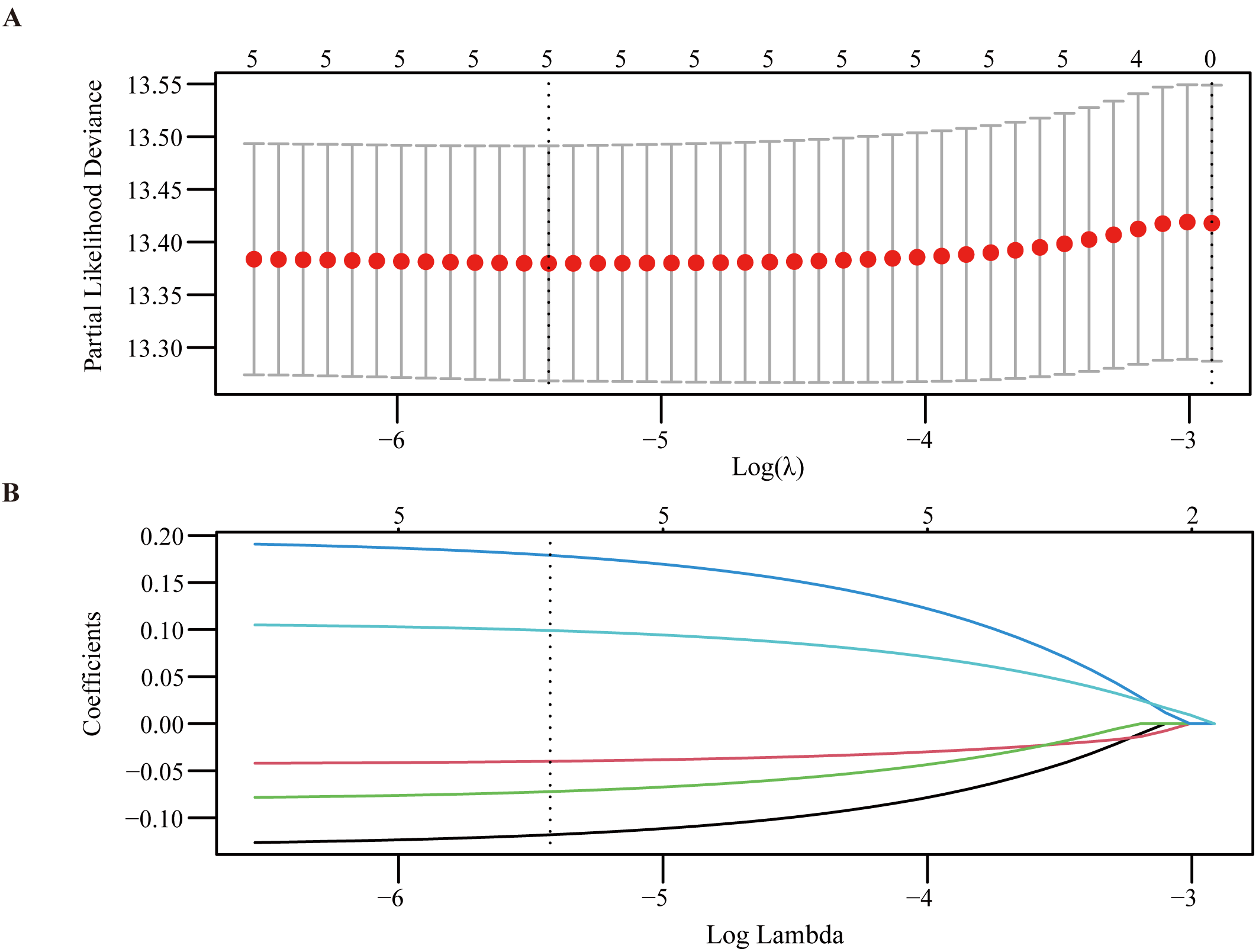

Supplement: Supplementary file 2 — Figure S2: Selection of feature genes based on LASSO regression analysis. (A) The optimal value of the parameter lambda was determined using tenfold cross‐validation. The lambda value at the left dotted line represents the minimum model deviation, while the lambda value at the right dotted line represents one standard deviation of the minimum model deviation. Values above the plot indicate the number of filtered feature genes. (B) Diagram showing the relationship between lambda and regression coefficients. Each gene is represented by a curve, and the lambda value corresponding to the optimal model performance is indicated by the dotted line. Five of the six genes were selected for subsequent analysis. [file CAM4-14-e71337-s001.tif]

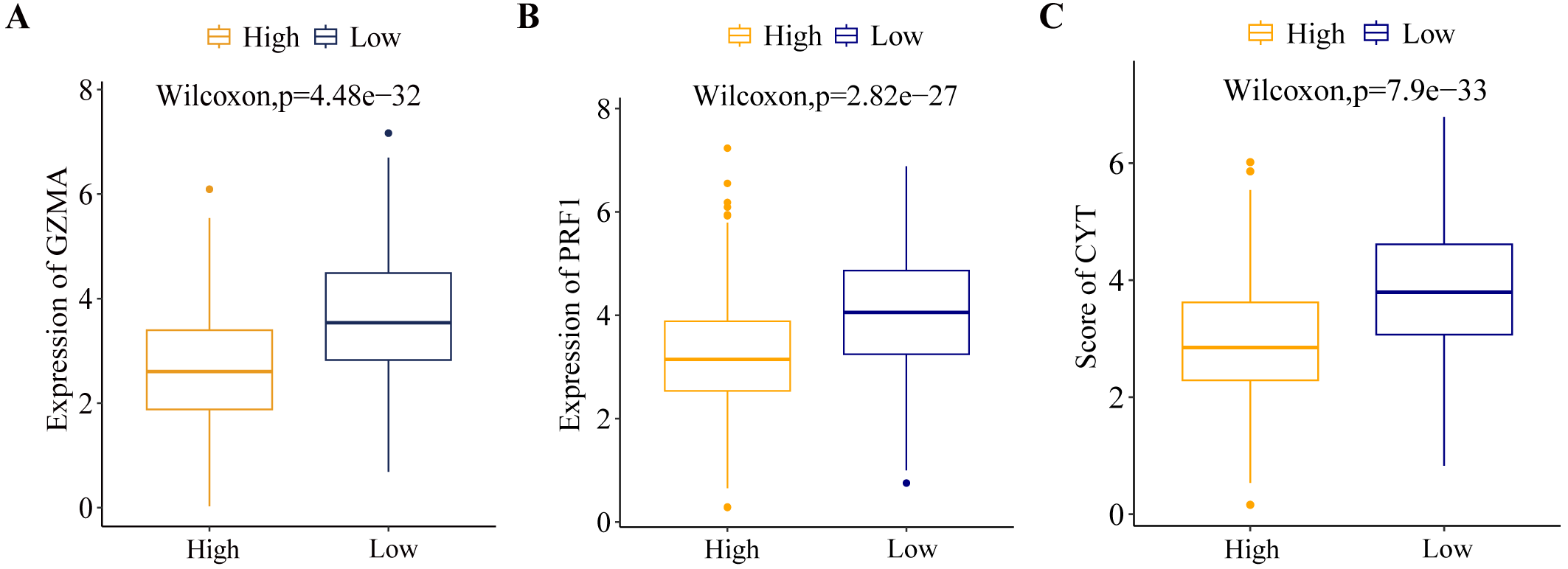

Supplement: Supplementary file 3 — Figure S3: Differential analysis of CYT scores. Elevated expression of PRF1 (A) and GZMA (B), along with a higher total CYT score (C), was observed in the low‐risk population. [file CAM4-14-e71337-s005.tif]
